# Haplotype and isoform specific expression estimation using multi-mapping RNA-seq reads

**DOI:** 10.1186/gb-2011-12-2-r13

**Published:** 2011-02-10

**Authors:** Ernest Turro, Shu-Yi Su, Ângela Gonçalves, Lachlan JM Coin, Sylvia Richardson, Alex Lewin

**Affiliations:** 1 Department of Epidemiology and Biostatistics, Imperial College London, Norfolk Place, London, W2 1PG, UK; 2 Ernest Gallo Clinic and Research Center, Department of Bioinformatics, University of California, San Francisco, 5858 Horton Street, Suite 200, Emeryville, CA 94608, USA; 3 European Bioinformatics Institute (EMBL-EBI), Wellcome Trust Genome Campus, Hinxton, Cambridge CB10 1SD, UK

## Abstract

We present a novel pipeline and methodology for simultaneously estimating isoform expression and allelic imbalance in diploid organisms using RNA-seq data. We achieve this by modeling the expression of haplotype-specific isoforms. If unknown, the two parental isoform sequences can be individually reconstructed. A new statistical method, MMSEQ, deconvolves the mapping of reads to multiple transcripts (isoforms or haplotype-specific isoforms). Our software can take into account non-uniform read generation and works with paired-end reads.

## Background

High-throughput sequencing of RNA, known as RNA-seq, is a promising new approach to transcriptome profiling. RNA-seq has a greater dynamic range than microarrays, which suffer from non-specific hybridization and saturation biases. Transcriptional subsequences spanning multiple exons can be directly observed, allowing more precise estimation of the expression levels of splice variants. Moreover, unlike traditional expression arrays, RNA-seq produces sequence information that can be used for genotyping and phasing of haplotypes, thus permitting inferences to be made about the expression of each of the two parental haplotypes of a transcript in a diploid organism.

The first step in RNA-seq experiments is the preparation of cDNA libraries, whereby RNA is isolated, fragmented and synthesized to cDNA. Sequencing of one or both ends of the fragments then takes place to produce millions of short reads and an associated base call uncertainty measure for each position in each read. The reads are then aligned, usually allowing for sequencing errors and polymorphisms, to a set of reference chromosomes or transcripts. The alignments of the reads are the fundamental data used to study biological phenomena such as isoform expression levels and allelic imbalance. Methods have recently been developed to estimate these two quantities separately but no approaches exist to make inferences about them simultaneously to estimate expression at the haplotype *and *isoform ('haplo-isoform') level. In diploid organisms, this level of analysis can contribute to our understanding of *cis *vs. *trans *regulation [1] and epigenetic effects such as genomic imprinting [2]. We first set out the problems of isoform level expression, allelic mapping biases and allelic imbalance, and then propose a pipeline and statistical model to deal with them.

### Isoform level expression

Multiple isoforms of the same gene and multiple genes within paralogos gene families often exhibit exonic sequence similarity or identity. Therefore, given the short length of reads relative to isoforms, many reads map to multiple transcripts (Table 1). Discarding multi-mapping reads leads to a significant loss of information as well as a systematic underestimation of expression estimates. For reads that map to multiple locations, one solution is to distribute the multi-mapping reads according to the coverage ratios at each location using only single-mapping reads [3]. However, this does not address the problem of inferring expression levels at the isoform level.

**Table 1:** Multi-mapping reads. Approximate proportion of reads mapping to multiple Ensembl transcripts or genes in human using 37 bp single-end or paired-end data obtained from HapMap individuals.

	37 bp single-end	37 bp paired-end
Multiple transcripts	78%	73%
Multiple genes	20%	10%

Essentially, the estimation of isoform level expression can be done by constructing a matrix of indicator functions *M_it _*= 1 if region *i *belongs to transcript *t*. The 'regions' may for now be thought of as exons or part exons, though we later define them more generally. Using this construction it is natural to define a model:

(1) $$X_{it}∼Pois(bs_{i}M_{it}\mu_{t}),$$

where *X_it _*are the (unobserved) counts of reads from region *i *of transcript *t*, *b *is a normalization constant used when comparing experiments, *μ_t _*is a parameter representing the expression of transcript *t *and *s_i _*is the *effective *length of region *i *(that is the number of possible start positions for reads in the region). This model can be fit using an expectation maximization (EM) algorithm, since the *X_it _*are unobserved but their sums across transcripts $k_{i}\equiv \sum_{t}X_{it}$ are observed.

This model has been used by [4] in their POEM software, with *i *representing exons. Their method does not use reads that span multiple exons or reads that map to multiple genes. The same model has been used in [5], with *i *representing exons or part exons, or regions spanning exon junctions, enabling good estimation of isoform expression within genes. They do not, however, include reads mapping to multiple genes. The RSEM method [6] employs a similar model, but models the probability of each read individually, rather than read counts. This method allows reads to come from multiple genes as well as multiple isoforms of the same gene. The modeling of individual reads allows RSEM to accommodate general position-specific biases in the generation of reads. However, two recent papers [7,8] have shown that deviations from uniformity in the generation of reads are in great part sequence rather than position-dependent for a given experimental protocol and sequencing platform. Furthermore, the computational requirements of modeling individual reads increasing proportionately with read depth, which, in the case of RSEM, is exacerbated further by the use of computationally intensive bootstrapping procedures to estimate standard errors. None of the above methods are compatible with paired-end data. A recently published method, Cufflinks [9], focuses on transcript assembly as well as expression estimation using an extension of the [5] model that is compatible with paired-end data. However, this method does not model sequence-specific uniformity biases and uses a fixed down-weighting scheme to account for reads mapping to more than one transcription locus, meaning that the abundances of transcripts in different regions are estimated independently.

### Allelic imbalance

Studies of imbalances between the expression of two parental haplotypes have mostly been restricted to testing the null hypothesis of equal expression between two alleles at a single heterozygous base, typically with a binomial test [1,2,10]. However, as transcripts may contain multiple heterozygotes, a more powerful approach is to assess the presence of a consistent imbalance across all the heterozygotes in a gene together. This has been done on a case-by-case basis using read pairs that overlap two heterozygous SNPs [11] while [12] propose an extension to the binomial test for detecting allelic imbalance that takes into account all SNPs and their positions in a gene. However, this approach, which is a statistical test rather than a method of quantifying haplotype-specific expression, assumes imbalances to be homogeneous along genes and thus does not take into account the possibility of asymmetric imbalances between isoforms of the same gene.

### Allelic mapping biases

Aligners usually have a maximum tolerance threshold for mismatches between reads and the reference. Reads containing non-reference alleles are less likely to align than reads matching the reference exactly, so genes with a high frequency of non-reference alleles may be underestimated. Ideally, aligners would accept ambiguity codes for alleles that segregate in the species (cf. Novoalign [13]), but no free software is currently able to do this. A possible workaround is to change the nucleotide at each SNP to an allele that does not segregate in the species, as has been proposed to remove biases when estimating allelic imbalance [10]. However, in the context of gene expression analysis, this leads to even greater underestimation of genes with many non-reference alleles and an increase in incorrect alignments to homologous regions. Instead, we propose aligning to a sample-specific transcriptome reference, constructed from (potentially phased) genotype calls.

### MMSEQ

In this paper we present a new pipeline, including a novel statistical method called MMSEQ, for estimating haplotype, isoform and gene specific expression. The MMSEQ software is straightforward to use, fully documented and freely available online [14] and as part of ArrayExpressHTS [15]. Our pipeline exploits all reads that can be mapped to at least one annotated transcript sequence and reduces the number of alignments missed due to the presence of non-reference alleles. It is compatible with paired-end data and makes use of inferred insert size information to choose the best alignments. Our method permits estimating the expression of the two versions of each heterozygote-containing isoform ('haplo-isoform') individually and thus it can detect asymmetric imbalances between isoforms of the same gene. Our software further takes into account sequence-specific deviations from uniform sampling of reads using the model described in [8] but can flexibly accommodate other models. We validate our method at the isoform level with a simulation study, comparing our results to RSEM's, and applying it to a published Illumina dataset consisting of lymphoblastoid cell lines from 61 HapMap individuals [16]. We validate our method at the haplo-isoform level by showing we can deconvolve the expression estimates of haplo-isoforms on the non-pseudoautosomal (non-PAR) region of the X chromosome using a pooled dataset of two HapMap males. We further apply our method to a published dataset of F_1 _initial and reciprocal crosses of CAST/EiJ (CAST) and C57BL/6J (C57) inbred mice [2] and demonstrate that MMSEQ is able to detect parental imbalance between the two haplotypes of each isoform.

## Results

### Overview of the pipeline

**Figure 1:**
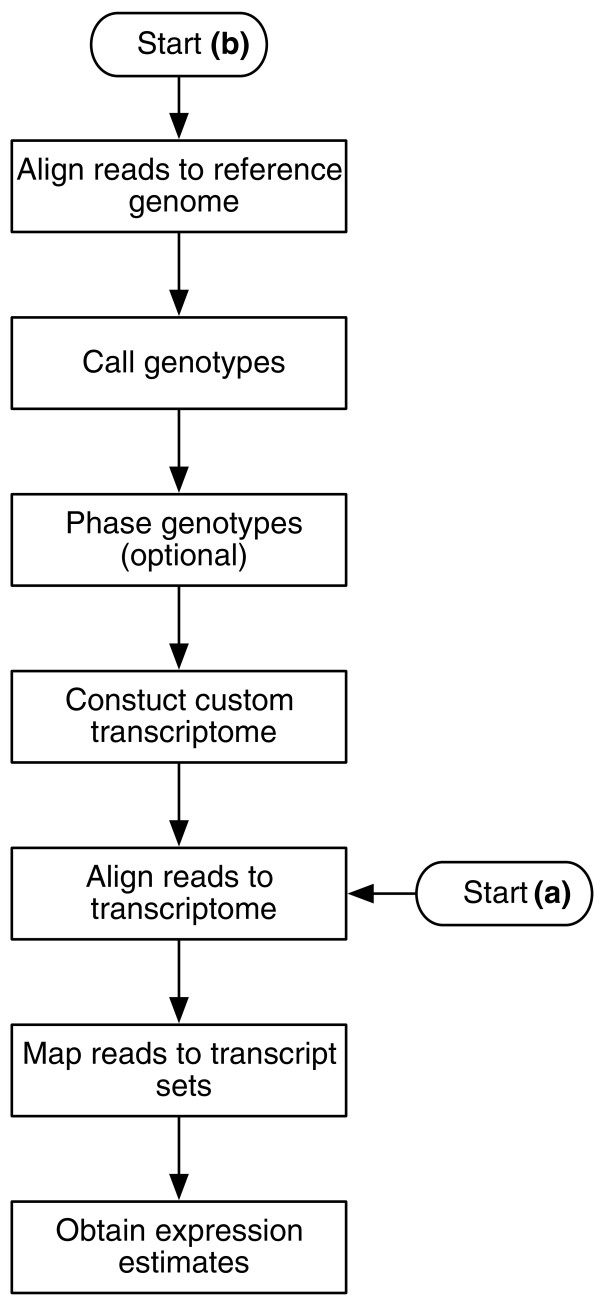
**Pipeline flow chart**. Flow chart depicting the steps in the pipeline and two main use cases. **(a) **expression estimation using a pre-defined transcriptome reference; **(b) **construction of a custom transcriptome reference from the data followed by expression estimation. Haplotype-specific expression can be obtained using a pre-defined transcript reference if the parental transcriptome sequences are known and recombination has no effect (for example in the case of an F_1 _cross of two inbred strains). If the standard (for example Ensembl) reference is used, then isoform-level estimates are produced. If a custom reference is constructed solely to avoid allelic mapping biases, the phasing of genotypes can be omitted and isoform-level estimates are produced. If the genotypes are phased, haplo-isoform estimation is performed.

The pipeline can be depicted as a flow chart with two different start positions (Figure 1):

(a) Expression estimation using alignments to a pre-defined transcriptome reference,

(b) Expression estimation using alignments to a transcriptome reference that is obtained from the RNA-seq data.

In case (a), the level of estimation (haplo-isoform or isoform) depends on whether the reference includes two copies of heterozygous transcripts. In case (b), it depends on whether the genotypes are phased. The most exhaustive use of the pipeline proceeds as follows. First, the reads are aligned to the standard genome reference using TopHat [17]. Then, genotypes are called with SAMtools pileup [18]. Genotypes are then phased with polyHap [19] using population genotype data to produce a pair of haplotypes for all gene regions on the genome. The standard transcriptome reference is then edited for each individual to match the inferred haplotypes. The reads are realigned to the individualized haplotype specific transcriptome reference with Bowtie [20], finding alignments for reads that originally failed to align due to having too many mismatches with the standard reference (approximately 0:3% more reads recovered, with some transcripts receiving up to 13% more hits, in the HapMap dataset [16]). Finally, our new method, MMSEQ, is used to disaggregate the expression level of each haplo-isoform.

### MMSEQ

#### Poisson model

We use the model in Equation 1 as a starting point for modeling gene isoforms and extend it to apply to haplo-isoforms. First, we employ a more general definition of 'region': each read maps to one set of transcripts, which may belong to the same gene or to various different genes, and which can have two versions, one containing the paternal and the other the maternal haplotype. These sets are labeled by *i*. Many reads will map to the exact same set, hence we can model reads counts (*k_i_*) for the set. The *M_it _*are defined very straightforwardly as the indicator functions for transcript *t *belonging to set *i*. The region length *s_i _*is the effective length of the sequence shared between the whole set. If the set of transcripts all belong to the same gene and haplotype, then *s_i _*may be the effective length of an exon or part exon. However, aligned reads often map to multiple genes equally well (Table 1) so the region need not correspond to an actual region on the genome. Using our definition of a region, the *s_i _*would be difficult to calculate given the sheer number of overlaps and regions, but in fact the *s_i _*are not needed in the calculation of the model (see Materials and Methods). Hence we have a model for read counts in which the data and fixed quantities (*k_i _*and *M_it_*) are calculated in a straightforward way, and which allows for reads mapping to multiple isoforms of the same or different genes in exons or exon junctions and to paternal and maternal haplotypes separately.

**Figure 2:**
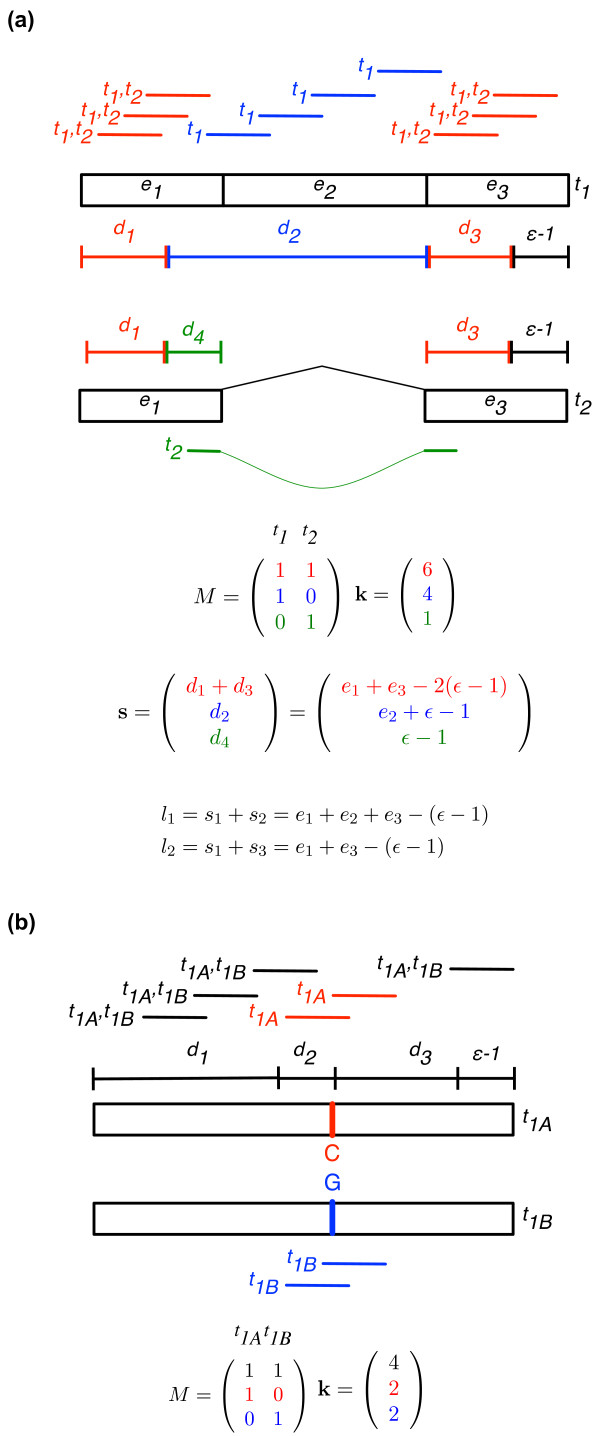
**MMSEQ data structures to represent read mappings to alternative isoforms and alternative haplotypes**. **(a) **Schematic of a gene with an alternatively spliced cassette exon. Each read is labeled according to the transcripts it maps to and placed along its alignment position. Reads that map to both transcripts, *t*_1 _and *t*_2_, are shown in red, reads that map only to *t*_1 _are shown in blue and the read that maps only to *t*_2 _is shown in green. Reads that align with their start positions in the regions labeled by *d*_1 _and *d*_3 _(in red) may have come from either transcript, reads with their start positions in *d*_2 _(in blue) can only have come from transcript 1, and reads with their start positions in *d*_4 _(in green) must be from transcript 2. Each row *i *of the indicator matrix *M *characterizes a unique set of transcripts that is mapped to by *k_i _*reads. There are three transcript sets: {*t*_1_, *t*_2_} (red), {*t*_1_} (blue) and {*t*_2_} (green). Exon lengths are *e*_1_, *e*_2_, *e*_3_. Hence *s*_1 _= *d*_1 _+ *d*_3_, *s*_2 _= *d*_2 _and *s*_3 _= *d*_4_. The effective length of transcript *t *is equal to the sum over the elements of **s **that have a corresponding 1 in column *t *of *M*, that is ∑*_i _s_i_M_it_*. It can be seen from the figure that these lengths are the sums of the exons minus read length (ϵ) plus one, as expected. (**b**)Schematic of a single-exon gene with a heterozygote near the center. Reads with starting positions in region *d*_2 _contain either the 'C' allele or the 'G' allele and thus map to either the haplo-isoform *t*_1*A*_, which has a 'C' or *t*_1*B*_, which has a 'G'. It is evident that the heterozygote acts like an alternative middle exon, and that the same model and data structures as in the alternative isoform schematic apply.

Without loss of generality, Figure 2a illustrates our formulation for a gene with an alternatively spliced cassette exon and Figure 2b illustrates it for a gene with a single heterozygous base. The heterozygote casts a 'shadow' upstream of length equal to the read length, which acts like an alternative middle exon. This is because reads with starting positions within the shadow cover the heterozygote and contain one of the two alleles, thus mapping to only one of the two haplotypes.

We now formulate a Poisson model for read counts from transcript sets:

(2) $$k_{i}∼Pois\left(bs_{i}\sum_{t}M_{it}\mu_{t}\right),$$

where *b *is a normalization constant, ∑*_t _M_it_μ_t _*is the total expression from the transcript set *i *and *s_i _*is the effective length of the region of shared sequence between transcripts in set *i*. Figure 2a shows how the *s_i _*can be calculated for the gene with a cassette exon. Note that the sum of lengths of all the regions shared by transcript *t *add up to its effective length (transcript length minus read length plus one for uniformly generated reads): ∑*_i _s_i_M_it _*= *l_t_*, so the transcript-set model is consistent with the usual Poisson model. Setting *l_t _*to the transcript length minus read length plus one is appropriate if a constant Poisson rate is assumed along all positions in a transcript: $r_{t}∼Pois(b\sum_{p=1}^{l_{t}}\mu_{t})∼Pois(bl_{t}\mu_{t})$, where *r_t _*is the number of reads originating from transcript *t *and the sum is over all possible starting read positions *p*. The non-uniformity of read generation demonstrated in [8], however, suggests a variable-rate Poisson model:

(3) $$r_{t}∼Pois\left(b\sum_{p=1}^{l_{t}}\alpha_{tp}\mu_{t}\right)∼Pois\left(b{\tilde{l}}_{t}\mu_{t}\right),$$

where ${\tilde{l}}_{t}$ is an adjusted effective length, referred to as the sum of sequence preferences (SSP) in [8]. We use their Poisson regression model to adjust the length of each transcript based on its sequence, but other adjustment procedures may be used instead. Briefly, the logarithm of the sequencing preference of each possible start position in a transcript is calculated as the sum of an intercept term plus a set of coefficients determined by the sequence immediately upstream and downstream of the start position. It would also be possible to integrate the method described in [7], which uses a weighting for reads based on the first seven nucleotides of their sequences, by applying this weighting in our calculation of *k_i_*. However, this approach does not incorporate the effects of the sequence composition on the reference upstream of the read start positions or further downstream than seven bases, and we thus prefer to use the [8] method instead. The normalization constant *b *is used to make lanes with different read depths comparable. We set *b *to the total number of reads (in millions) and measure transcript lengths in kilobases, which means the scale of the expression parameter *μ_t _*is equivalent to RPKM (reads per kilobase per million mapped reads) described in [3]. In downstream analysis, a more robust measure can be used, such as the library size parameter suggested by [21].

The only unknown parameters in the model are the *μ_t_*. The observed data are the *k_i _*and the matrix *M *and effective transcript lengths *l_t _*are known. In principle the effective lengths of the transcript sets *s_i _*can be calculated, but in fact, they are not needed (see Materials and Methods).

#### Inference

The maximum likelihood (ML) estimate of *μ_t _*cannot be obtained analytically, so instead we use an expectation maximization (EM) algorithm to compute it, an approach also taken by [4,6] for isoforms. After convergence of the algorithm, we output the estimates of *μ_t _*and refer to them as MMSEQ EM estimates.

The usual approach to estimating statistical standard errors of ML estimators requires inversion of the observed information matrix. When analyzing the expression of thousands of transcripts, the high dimensionality of the observed information matrix and the possibility of identical columns due to gene homology make this approach impracticable. Bootstrapping may also be used to estimate errors, as in [6], but it is a computationally intensive method requiring repeated runs of the EM algorithm. Instead we use a simple Bayesian model with a vague prior on *μ_t_*. As before, we use the augmented data reads per region and transcript, *X_it_*. The full model is:

(4) $$X_{it}|\mu_{t}\;∼Pois(bs_{i}M_{it}\mu_{t}),$$

(5) $$\mu_{t}∼Gam(\alpha ,\beta ).$$

Again, the only lengths needed are the *l_t_*. The conjugacy of the Poisson-Gamma model makes the sampling fast and straightforward as the full conditionals are in closed form (see Materials and Methods). We use the final EM estimate of the *μ_t _*as the initial values for the Gibbs sampling. We then produce samples from the whole posterior distributions of the *μ_t _*and calculate the sample means and their respective Monte Carlo standard errors (MCSE), which take into account the autocorrelations of the samples [22]. We set the hyperprior parameters to *α *= 1.2 and *β *= 0.001, producing a vague prior on the *μ_t _*that captures the well-known broad and skewed distribution of gene expression values. We output the means of the Gibbs samples of *μ_t_*, which we refer to as MMSEQ GS estimates. As we shall show, the regularization afforded by the Bayesian algorithm produces estimates with a lower error than the MMSEQ EM estimates. Moreover, it can readily be shown that for transcript with low coverage, the ML estimate is often zero, even though this is likely to be an underestimate of the expression. For example, suppose there exist two equally-expressed haplo-isoforms differing by only one heterozygote. Under the assumption of uniform sampling of 0.01 reads per nucleotide for both haplo-isoforms, if the read length is 35, then the probability of observing a read containing one allele but no reads containing the other allele is fairly high (2(1-*e*^-0.35^)*e*^-0.35 ^≃ 0.42). The ML estimate of the haplo-isoform with the unsampled allele under this scenario is zero while the ML estimate of the haplo-isoform with the sampled allele is overestimated. With Gibbs sampling, on the other hand, this effect is tempered by the Gamma prior. The MMSEQ GS estimates are thus our preferred expression measures.

#### Best mismatch stratum filter

While a read may align to multiple transcripts, not all alignments may be equally reliable. We therefore filter out all alignments that do not have the minimal number of mismatches for a given read or read pair (similar to the --strata switch in Bowtie, but compatible with paired as well as single end data). In the case of paired-end data, the number of mismatches from both ends is added up to determine the 'mismatch stratum' of a read pair. This filter is crucial in order to correctly discriminate between the two versions of an isoform at a heterozygous position, since reads from one haplotype also match the alternative haplotype with an additional mismatch. The stratum filter thus ensures that reads are properly assigned to the correct haplotype.

#### Insert size filter for paired-end data

For paired-end data, both reads in a pair must align to a transcript for the mapping to be considered. If the fragments are sufficiently large, the alignments may span three exons and align to transcripts that both retain and skip the middle exon. However, the alignment with an inferred fragment size (also called insert size) that is nearer to the expected insert size from the fragmentation protocol, is more likely to be correct. We exploit this information by applying an insert size filter to alignments in the best mismatch stratum for each read. If an alignment's insert size is nearer than *x *bp (for example equivalent to one standard deviation) away from the expected insert size, then all other alignments for that read with an insert size greater than *x *bp away from the expected insert size are removed. This filter can be thought of as an extension of mismatch-based filtering for reporting only alignments with moderately high probability of being true. Although full probabilistic modeling is more principled, filtering is a commonplace approach to reducing alignment candidates for each read to a set that can be dealt with pragmatically. For the HapMap dataset, mistakes in the protocol resulted in two distributions of insert sizes within some samples, so we omitted this filter.

#### MMSEQ output

The *mmseq *program produces three files each containing EM and GS expression estimates with associated MCSEs. The first file provides estimates at the transcript/haplo-isoform level, the second file provides aggregate estimates for sets of transcripts that have been amalgamated due to having identical sequences (and therefore indistinguishable expression levels), and the third file aggregates transcript estimates into genes, thus providing gene-level estimates. Homozygous transcripts are aggregated together, whereas heterozygous transcripts are aggregated separately to produce 'haplo-gene' level estimates. With respect to transcripts that have identical sequences and hence indistinguishable and unidentifiable expression levels, the posterior samples exhibit high variance and strong anti-correlation but the sum of their expression can be precisely estimated (Additional file 1). We therefore recommend use of the amalgamated estimates.

### Performance and scalability

The performance of the EM and Gibbs algorithms is determined principally by the size of the *M *matrix, which is bounded by the total number of known transcripts and the total number of combinations of transcripts that share sequence. Marginal increases in the total number of observed reads do not result in commensurate increases in the size of *M*, because additional reads tend to map to transcript sets that have been mapped to by previous reads (Table 2). Consequently, the *mmseq *program exhibits economies of scale which allow it to cope with future increases in throughput. This contrasts with the RSEM method, which represents each read separately in their indicator matrix that maps reads to isoforms [6].

**Table 2:** *mmseq *performance. Performance of the *mmseq *program on subsets of different sizes of the HapMap paired-end dataset.

Read pairs (millions)	Dimension of *M*	Runtime (seconds)
1	63,924 × 68,666	507
2	84,417 × 75,649	541
3	97,576 × 79,035	746
4	107,344 × 81,289	793
8	134,489 × 86,528	1,047
16	166,100 × 91,023	1,204

Where necessary in order to obtain a large enough dataset, reads from multiple lanes of the same individual were pooled. The program exhibits economies of scale because the dimension of *M *increases more slowly than the number of reads.

### Correction for non-uniform read sampling

**Figure 3:**
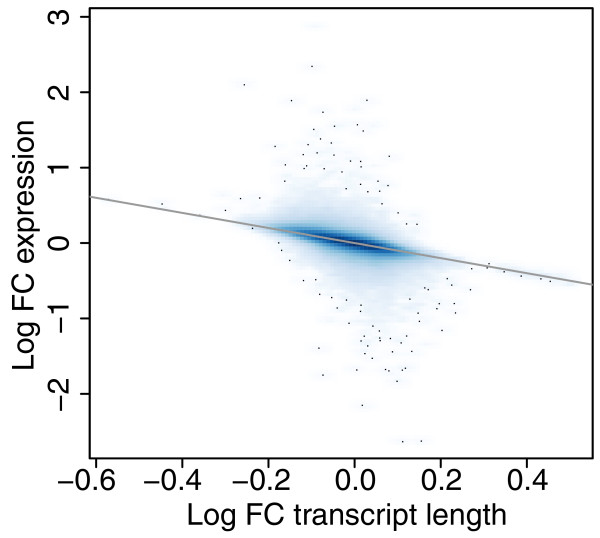
**Impact on expression of transcript lengths adjustment**. Smooth scatterplot of the log fold change in transcript length after adjusting for non-uniform read generation vs. the log fold change in expression. The hundred transcripts in the lowest density regions are shown as black dots. Changes in the expression estimates tend to be inversely proportional to adjustments to the lengths but for some transcripts even a small change in the length has a significant impact on the expression estimate.

We have assessed the effect of applying the Poisson regression [8] correction for non-uniform sampling using read data from three Illumina Genome Analyzer II (GAII) lanes from the HapMap dataset [16] (described below). Two of the samples were from the same run (ID 3125) and a third from a separate run (ID 3122). We obtained Poisson regression coefficients for 20 bases upstream and downstream of each possible start position using the first 10 million alignments for each lane. The regression model was fitted using only the most highly expressed transcripts, as these have the best signal-to-noise ratio [8]. Specifically, from the 500 transcripts with the highest average number of nucleotides per position, we selected a subset containing only one transcript per gene so as to avoid double-counting of sequence preferences. As shown in Additional file 2, the coefficients are highly stable across both lanes and runs. The time-consuming task of calculating adjusted transcript lengths separately for each lane is therefore unnecessary. Instead, our software can reuse the adjusted transcript lengths calculated from one sample when analyzing other samples. Variations in the Poisson rate from base to base tend to average out over the length of each transcript, and thus the adjustments to the lengths are generally slight (Additional file 3). As expected from the Poisson model (Equation 3), changes in the expression estimates (estimates of *μ_t_*) tend to be inversely proportional to adjustments to the lengths. Nevertheless, as transcripts sharing reads may be adjusted in opposite directions, for some transcripts even a small change in the length has a significant impact on the expression estimate (Figure 3).

### Simulation study of isoform expression estimation

**Figure 4:**
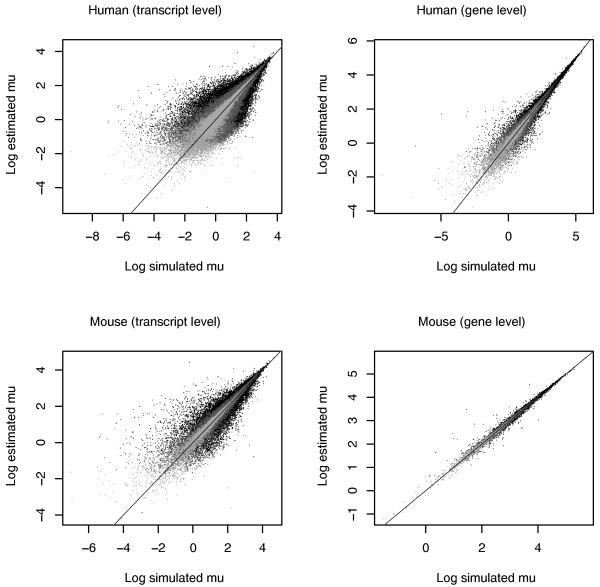
**Isoform-level simulation scatterplots**. Scatterplots comparing log-scale simulated vs. estimated RPKM expression values for human and mouse at the transcript and gene levels. Estimates with MCSE greater than the median are shown in black, lower than the median but higher than the bottom 10% are shown in dark grey and lower than the bottom 10% are shown in light grey.

We simulated reads from human and mouse Ensembl cDNA files under the assumption of uniform sampling of reads and ran the MMSEQ workflow. We found good correlation between simulated and estimated expression values and between dispersion around the true values and estimated MCSEs. We did however observe a small upward bias in our estimates of transcripts with low expression levels, attributable to our use of the mean to summarize highly skewed distributions. We evaluated our gene-level estimates by summing over the isoform components within each gene. As anticipated, we obtained more precise estimates for genes than for transcripts (Figure 4).

**Figure 5:**
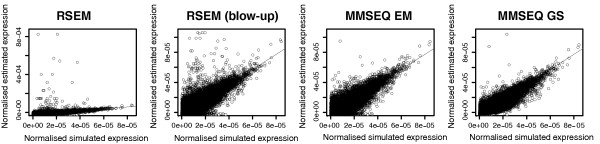
**Scatterplots comparing RSEM with MMSEQ**. Scatterplots comparing simulated vs. estimated normalized expression values from RSEM, MMSEQ EM and MMSEQ GS for a simulated human dataset. The second RSEM plot from the left is a blown up version of the plot on the far left so that the y-axis covers the same range as the MMSEQ plots on the right.

We also observed better estimates for mouse, which has 45,452 annotated transcripts, than for human, which has higher splicing complexity manifested in 122,636 annotated transcripts (Figure 5). Transcripts may be connected to other transcripts via reads that align to regions shared by isoforms of the same gene or to different genes with sequence homology. The complexity of the graph that connects transcripts with each other reflects the ambiguity in the assignment of reads to transcripts and thus the errors in our estimates. A bar plot of the number of transcripts that each transcript is connected to in human and mouse demonstrates a significant difference in complexity between the annotated transcriptomes of the two species (Additional file 4).

### Comparison of isoform expression estimation between MMSEQ and RSEM

Like MMSEQ, the RSEM method [6] makes use of all classes of reads to estimate isoform expression. The authors have shown an improvement of their method for gene-level estimation over strategies that discard multiply aligned reads or allocate them to mapped transcripts according to the coverage by single-mapping reads (as in [3]). However, isoform-level results for their method have not been assessed. We obtained RSEM estimates for Ensembl transcripts using our simulated human sequence dataset for the purposes of comparison.

We scaled our simulated and estimated expression values to add up to one in order to make them comparable to RSEM's fractional expression estimates. We found that RSEM and MMSEQ EM are comparable but, unlike the MMSEQ EM algorithm, RSEM tended to overestimate some medium-expression transcripts. Both the RSEM and MMSEQ EM algorithms tended to underestimate some low-expression transcripts, pushing them very close to zero and thus producing very large errors on the log scale. This was avoided by the regularization of the Gibbs algorithm, which produced tighter estimates and only overestimated slightly some very lowly expressed transcripts (Figure 5 and Additional file 5), showing the benefits of using the whole posterior distribution of *μ_t _*to estimate expression rather than a maximization strategy.

### Isoform-level application to the HapMap dataset

**Figure 6:**
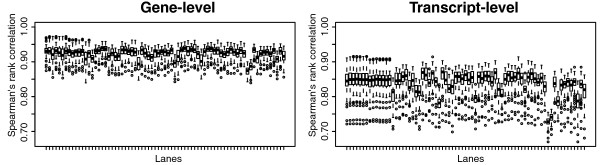
**Rank correlation box plots in the HapMap dataset**. Boxplots of pairwise Spearman's rank correlation between expression values in the HapMap dataset. The first and second sets of seven boxplots correspond to technical replicates while the remaining boxplots correspond to different CEU individuals.

The HapMap paired-end Illumina GAII dataset [16] consists of 73 lanes: 7 lanes for the same Yoruban individual, another 7 lanes for the same CEU individual and the remaining 59 lanes each for different CEU individuals. The authors assessed exon-count correlations between the lanes. Here we look at transcript and gene-level correlations. We analyzed the data using the MMSEQ pipeline, aligning approximately 75% of reads to Ensembl human reference transcripts. The average rank correlation was 0.92 and 0.84 respectively at the gene and transcript level (Figure 6). When comparing identical samples at the gene level the rank correlation ranged from 0.96 to 0.97 for the Yoruban individual and from 0.92 to 0.97 for the CEU individual. At the transcript level, the ranges were 0.91 to 0.92 and 0.90 to 0.91 for the Yoruban and CEU individuals respectively. The transcript-level values are comparable to exon-count correlations found by [16]. Both are lower than the gene-level correlation, as might be expected due to the inclusion of within-gene variance.

Although the ordering of transcripts and genes was broadly maintained even between lanes belonging to different individuals and runs, we found a striking contrast in the distribution of expression values between lanes of the same individual and lanes of different individuals (Additional file 6). The consistency of expression values for lanes of the same individual indicates that the technical replicability of the Illumina GAII sequencer is extremely high and therefore that the variation observed between lanes from different individuals is mostly a reflection of biological variability. This is in line with previous research showing that sequence count data follow a negative binomial distribution in biological replicates and a Poisson distribution in technical replicates [21]. As such, we expect the variance of our estimates to be proportional and greater than proportional to the expression values for technical and biological replicates respectively. This is indeed borne out both at the gene and transcript level (Additional file 7) and corroborates the need to take into account extra variability for highly-expressed transcripts in differential expression analysis with biological replication (see Discussion).

### Validation of haplo-isoform deconvolution

The non-pseudoautosomal region (non-PAR) of the X chromosome in human males is haploid, and thus the alleles in that region can be called directly without the need for phasing. We validated our method for deconvolving expression between two haplotypes of the same isoform as follows. We used the RNA-seq data of two males from the HapMap data (NA12045 and NA12872) to call their haplotypes. We identified 117 isoforms on the non-PAR of the X chromosome that differed between the two individuals. We created custom transcriptome references for each of the two males, containing their individual versions of the 117 isoforms. We then created a third hybrid reference containing two copies of the 117 isoforms, one matching the haplotype of one male and the second matching the haplotype of the other. This hybrid reference mimics the case of a female with two X chromosomes with unknown expression of the two parental copies of each isoform. We obtained individual expression estimates of the 117 isoforms using the separate transcriptome references in each male and compared them with estimates obtained by aligning a dataset pooled from the data of both males to the hybrid reference. Although the original correlation between the two males was 0.85, the correlation between the individual estimates and the deconvolved estimates was 0.96 and 0.98, showing MMSEQ is capable of disaggregating the expression from paternal and maternal isoforms (Additional file 8).

**Figure 7:**
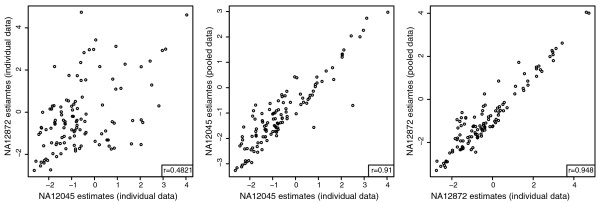
**Scatterplots of log expression estimates from individual and pooled data with read removal**. Left: scatterplot of log expression estimates of male NA12045 vs. NA12872 obtained from individual datasets where reads were removed from subsets of genes to decrease the correlation between the two individuals. Center: scatterplot of log expression estimates of male NA12045 obtained from the individual vs. pooled data. Right: scatterplot of log expression estimates of male NA12872 obtained from the individual vs. pooled data.

To test whether MMSEQ is able to recover greater imbalances than found naturally between the two male individuals, we divided the genes of the 117 isoforms that are heterozygous in the hybrid reference into three equal-sized groups. For one group, we artificially removed 90% of the reads hitting one male and, for another group, we artificially removed 90% of the reads hitting the other male. This reduction of reads mimics what would be observed if more extreme imbalances existed. We thus reduced the correlation between the log expression of the two males from 0.85 to 0.48. Despite this large imbalance, there was a correlation of 0.91 and 0.95 between the individual and the deconvolved estimates obtained from the pooled dataset (Figure 7), showing that MMSEQ is able to accurately disaggregate haplotype-specific expression in the presence of large imbalances.

### Demonstration of haplo-isoform expression estimation using an F_1 _hybrid mouse brain dataset

We have applied MMSEQ to a published murine embryonic day 15 RNA-seq dataset of CAST/C57 initial (F_1_i) and reciprocal (F_1_r) crosses [2]. Each RNA sample was a pool from four individuals. The C57 reference transcriptome used by the authors is available from the UCSC Genome Browser [23]. The authors called SNPs by aligning reads from the CAST samples to the C57 reference. We created a CAST reference transcriptome by changing alleles in the C57 reference sequences according to those SNP calls. The two references were combined in a hybrid reference containing two entries for isoforms that differed in sequence between C57 and CAST. Thus there is a one-to-one mapping between SNPs called in the parents and heterozygotes in the hybrids. The data consist of 152 and 159 million 36 bp Illumina GAII reads for F_1_i and F_1_r respectively.

**Figure 8:**
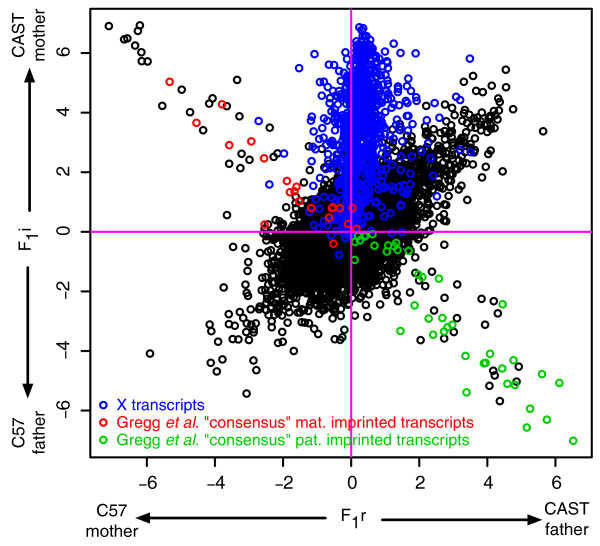
**Reciprocal vs. initial cross, highlighting 'consensus' imbalanced isoforms and X transcripts**. Scatterplot of log fold changes between haplo-isoforms in the reciprocal (F_1_r) and the initial (F_1_i) cross, highlighting X transcripts in blue, isoforms termed 'consensus' maternally imprinted in red and 'consensus' paternally imprinted in green. 'Consensus' imprinted genes were chosen by [2] as those with more than two heterozygotes exhibiting imbalance in favor of the same parental sex, at least one of which was significant in a *χ*^2 ^goodness-of-fit test with a *P-value *threshold of 0.05.

A scatterplot of the CAST/C57 differential expression between F_1_i and F_1_r crosses reveal a clear clustering of points into three groups (Figure 8). Firstly, the points on the upper-left to lower-right diagonal correspond to transcripts which show imbalance towards the parent of origin, suggesting they are imprinted. Those on the upper-left quadrant and bottom-right quadrant correspond to maternally and paternally imprinted transcripts respectively. Transcripts termed 'consensus imprinted' by [2] are highlighted in color. These were defined arbitrarily by the authors as transcripts with more than two heterozygotes exhibiting imbalance in favor of the same parental sex, at least one of which was significant in a *χ*^2 ^goodness-of- fit test with a *P-value *threshold of 0.05.

We also identified a clustering of transcripts that exhibited CAST overexpression in the F_1_i hybrids but approximately balanced expression in the F_1_r's. We identified the cluster as consisting wholly of transcripts on the X chromosome (Additional file 9), which suggests that the initial crosses were male and the reciprocal crosses female. The sexes of the hybrid mice are in fact unknown. There was a slight skew in favor of the CAST strand in the reciprocal crosses. We think it is unlikely that this was due to mapping biases, since the CAST reference was produced from SNP calls against the C57 reference and was thus of lower quality, so any mapping bias would be expected to be in favor of C57. Moreover, [24] found a similar skew in adult samples of the same crosses. It is possible that the skew is the result of a selective bias in favor of C57 X-inactivated cells [25], possibly caused by one or more of the three mutations on the X-inactivation transcript (UCSC ID uc009tzp.1) or mutations in its promoter region.

The third grouping in the plot is on the lower-left to upper-right diagonal. These transcripts demonstrate consistent CAST/C57 differential expression regardless of the sex-strain combination of the parents, and are thus indicative of *cis *regulation.

One advantage of MMSEQ is that imbalances are assessed at the transcript level rather than for individual SNPs. Thus it is not necessary to set arbitrary thresholds on the numbers of heterozygotes or the magnitude and significance of the imbalances to make claims about transcript-level imbalances. Indeed, some of the transcripts that contain one or more heterozygote with a significant *P-value *but were not classified as 'consensus imprinted' by [2] are clearly shown to be imprinted by our results. Note however that 27 transcripts had significant heterozygotes with imbalances in opposing directions, demonstrating that it is not always appropriate to generalize from a single locus to make claims of imbalance at the transcript level (Figure 8 and Additional file 10).

**Figure 9:**
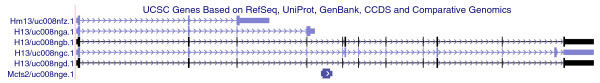
**Isoform structures of *H13 *and *Mcts2***. Labeled graphical depiction of *H13 *and *Mcts2 *UCSC isoform structures.

For genes containing heterozygotes with opposing imbalances, one approach is to scan the transcript annotations to identify isoform structures consistent with the observed SNP positions and imbalances. This approach was taken by [2], who defined these genes as 'complex' as long as at least one SNP was significant. An example of a complex gene is *H13*, which has two short isoforms and three longer isoforms with several additional exons towards the 3' end (Figure 9). The short isoforms contained heterozygotes with a paternal bias in their 3' exons while the heterozygotes on the 3' and intermediary exons of the longer isoforms had a maternal bias (cf. Figure S9 of [2] for a SNP-by-SNP visualization of the results of their preoptic area F_1 _samples). Using MMSEQ, we were able to discern this effect by direct quantification of haplo-isoforms. The two short isoforms were clearly imbalanced towards the paternally inherited haplotype while two of the long isoforms were clearly imbalanced towards the maternally inherited haplotype. An additional gene within the boundaries of *H13*, *Mcts2*, was also found to be paternally overexpressed (Table 3). By exploiting the data and annotation simultaneously, MMSEQ can be used to detect opposing imbalances between isoforms of the same gene directly.

**Table 3:** MMSEQ estimates for *H13 *and *Mcts2 *isoforms in F_1 _hybrid samples. MMSEQ estimates for each haplotype and isoform of *H13 *and *Mcts2 *of the initial and reciprocal crosses are shown.

	Mother		Father	
	CAST*_i_*	C57*_r_*	C57*_i_*	CAST*_r_*
uc008nfz.1	1.26	1.63	9.61	9.17
uc008nga.1	1.29	3.58	7.68	7.51
uc008ngb.1	12.97	9.81	0.94	0.39
uc008ngc.1	13.63	10.51	1.10	1.08
uc008ngd.1	0.22	0.18	0.30	0.13
uc008nge.1	2.01	4.20	11.29	14.66

The two short isoforms of *H13 *(uc008nfz.1 and uc008nga.1) were found to be paternally imprinted, while the longer isoforms uc008ngb.1 and uc008ngc.1 were found to be maternally imprinted. The long isoform uc008ngd.1 was estimated to be close to absent. The short *Mcts2 *gene (uc008nge.1), located within the boundaries of *H13*, was found to be paternally overexpressed.

## Discussion

We have presented a pipeline and statistical method that can disaggregate expression between isoforms and even between the two haplotypes of each isoform within an individual. MMSEQ produces improved isoform estimates compared to RSEM for medium to low expression transcripts, is more scalable, and estimates standard errors more efficiently. Furthermore, our principled approach to haplo-isoform quantification obviates the need for ad-hoc interpretations of SNP-by-SNP imbalances in terms of transcripts. Two aspects of our method, however, deserve further discussion.

### Transcript discovery

MMSEQ aims to quantify the abundance of known transcripts, and as such relies on the comprehensiveness of the transcriptome's annotation. It is usually possible to align a very large proportion of the reads to Ensembl transcripts (approximately 75% in the HapMap study using Ensembl version 56). However, samples may contain previously unobserved genes or isoforms. MMSEQ can in such cases work in tandem with transcript discovery methods by adding newly predicted isoform sequences to the reference transcript FASTA file and using it in the alignment and mapping steps of the MMSEQ workflow.

### Modeling biological variability

The Poisson distribution captures technical variability arising in repeated sequencing experiments with the same biological sample. The true expression value is, in effect, fixed by the experiment, and the only source of variability arises from measurement error and mapping uncertainty. However, between biological replicates such as different individuals in the HapMap study, there is, additionally, variability of a biological origin. As has been previously reported, this results in expression values between replicates that show overdispersion, captured, for example, by a negative binomial distribution [21].

Here we have focused on the problem of estimating the posterior distribution of expression values independently per sample. Nevertheless, it would be possible to add a further level to our Bayesian model to capture overdispersion across samples flexibly. For example, if exchangeable Gamma priors are set on the *μ_t_*, a suitable negative binomial model can be induced.

### Phasing with paired-end data

In this work, we have phased genotype calls obtained from SAMtools pileups - an approach that works well with both single and paired-end data. However, in the case of paired-end data, the haplotypes observed directly at multiple SNPs spanned by overlapping read pairs could be used to increase confidence in the phasing calls. Although incorporating this information would benefit phasing estimates only for some sets of SNPs, we believe it is a worthwhile area of future research. As phasing is a distinct step in our pipeline, improved methodologies can be integrated flexibly as they become available.

## Conclusions

RNA-seq is a promising and rapidly developing technology that provides sequence and expression intensity information of a sample in a single experiment. We have presented a novel pipeline and fast, scalable methodology to estimate expression of diploid organisms at the haplotype, isoform and gene levels. This allows researchers to go beyond allele-specific expression analysis and assess imbalance between paternal and maternal copies of isoforms, which in turn may be compared to differential isoform expression between individuals. We have shown that our method is able to deconvolve the expression of transcripts on each of two X chromosomes from human males in a pooled dataset, and that it can be successfully applied to detect genomic imprinting and *cis*-regulated transcripts in mouse hybrids. Our method retains reads that emanate from junctions as well as wholly within exons, models alignments to multiple transcripts, potentially across genes, exploits insert size information in paired-end data to choose the best alignments and flexibly incorporates corrective models for non-uniform read sampling. The pipeline, the MMSEQ software and related documentation are freely available online [14].

## Materials and methods

### Expectation maximization

We augment the data with the reads per region and transcript, *X_it_*, where Σ*_t _X_it _*= *k_i _*and use the Poisson approximation for the augmented data likelihood:

(6) $$X_{it}∼Pois(bs_{i}M_{it}\mu_{t}).$$

The distribution of the augmented data conditional on the observed data and the parameters is multinomial:

(7) $$\left\{X_{i1},...,X_{in}\right\}|\{\mu_{1},...,\mu_{t}\},k_{t}\sim Mult\left(k_{i},\frac{M_{i1}\mu_{1}}{\sum_{t}M_{it}\mu_{t}},...,\frac{M_{in}\mu_{n}}{\sum_{t}M_{it}\mu_{t}}\right)$$

(8) $$\Rightarrow E(X_{it}|k_{i},\mu_{t}^{\left(p\right)})=\frac{k_{i}M_{it}\mu_{t}^{\left(p\right)}}{\sum_{t^{\prime }}M_{it^{\prime }}\mu_{t^{\prime }}^{\left(p\right)}}.$$

The derivative of the expected Poisson log likelihood over *X *given *k *and *μ*^(*p*) ^with respect to *μ_t _*is linear in X, and hence

(9) $$argmaxE_{X|k,\mu^{\left(p\right)}}\log L(\mu ;X,b,M,s)=\arg\max\log L(\mu ;E(X|k,\mu^{\left(p\right)}),b,M,s).$$

The EM algorithm can be thus be expressed as repeatedly updating the $\mu_{t}^{\left(p\right)}$ at each iteration *p *using a form of the Poisson ML estimator in which the *X_it _*have been substituted with $E(X_{it}|k_{it},\mu_{t}^{\left(p\right)})$:

(10) $$\mu_{t}^{\left(p+1\right)}\leftarrow \frac{\sum_{i}X_{it}^{\left(p\right)}}{bl_{t}},$$

(11) $$\text{where}\;\frac{\sum_{i}X_{it}^{\left(p\right)}}{bl_{t}}=\frac{\mu_{t}^{\left(p\right)}}{bl_{t}}\sum_{i}\frac{k_{i}M_{it}}{\left(\sum_{t^{\prime }}M_{it^{\prime }}\mu_{t^{\prime }}^{\left(p\right)}\right)},$$

which converges to the ML estimate of *μ_t_*. To initialize the algorithm, we set *μ*^(0) ^equal to $\frac{1}{bl_{t}}\sum_{i}\frac{M_{it}k_{i}}{\Sigma_{t^{\prime }}M_{it^{\prime }}}$, which is equivalent to distributing *k_i _*evenly between cells of *X_i _*where *M_it _*is one. For a given region *i*, the probability of reads being allocated to a given transcript depends only on the *μ_t _*and not on *s_i _*(as the region is the same length on all transcripts). Hence, the *s_i _*do not appear in the update steps.

### Bayesian model and Gibbs sampling

As before, we use the augmented data reads per region and transcript, *X_it_*. The full model is:

(12) $$X_{it}|\mu_{t}∼Pois(bs_{i}M_{it}\mu_{t}),$$

(13) $$\mu_{t}∼Gam(\alpha ,\beta ).$$

The full conditionals are:

(14) $$\left\{X_{i1},...,X_{it}\right\}|\{\mu_{1},...,\mu_{t}\},k_{i}\sim Mult\left(k_{i},\frac{M_{i1}\mu_{1}}{\sum_{t}M_{it}\mu_{t}},...,\frac{M_{in}\mu_{n}}{\sum_{t}M_{it}\mu_{t}}\right),$$

(15) $$\mu_{t}|\{X_{1t},...X_{mt}\}\sim Gam\left(\alpha +\sum_{i}X_{it},\beta +bl_{t}\right).$$

Again, the *s_i _*are not needed as they are absent from the full conditionals.

### TopHat settings

Gapped alignment to the genome is performed with TopHat. We use a GFF file (specified with -G) based on the Ensembl annotation. We set --no-novel-juncs, --min-isoform-fraction 0.0 and --min-anchor-length 3. The expected inner distance between mate pairs is specified with the -r switch.

### SAMtools pileup settings

Genotypes output by SAMtools pileup were filtered using samtools.pl varFilter with default options and setting a minimum Phred-scaled probability of the genotype being identical to the reference ('SNP quality') threshold of 20.

### Bowtie settings

Alignment to the transcriptome with Bowtie is performed with the -a --best switches, which ensure all the best alignments in terms of mismatches are produced. Additionally, we recommend using --strata to output only alignments with the minimum number of mismatches, although it currently has no effect on paired-end data. The minimum and maximum insert sizes should be set appropriately with the -I and -X switches respectively, as should --norc/--nofw for stranded protocols.

## Abbreviations

CEU: Utah residents with ancestry from northern and western Europe; EM: expectation maximization; GAII: Genome Analyzer II; GS: Gibbs sampling; Haplo-isoform: haplotype-specific isoform; MCSE: Monte Carlo standard errors; ML: maximum likelihood; PAR: pseudo-autosomal region; RPKM: reads per kilobase per million mapped reads; SNP: single nucleotide polymorphism; UCSC: University of California: Santa Cruz.

## Authors' contributions

ET and AL developed the statistical model with the advice of SR and drafted the manuscript. ET implemented the MMSEQ software and ran validation experiments. SS and ET implemented the haplo-isoform pipeline and ran validation experiments. LC proposed and helped develop the EM algorithm and supervised the haplo-isoform validation experiment. AG proposed the application to mouse crosses and provided guidance on RNA-seq analysis. AG and ET applied the method to the mouse brain dataset. AL and SR supervised the project. AG, LC, SR and SS reviewed and revised the manuscript. All authors have read and approved the final manuscript.

## Supplementary Material

Additional file 1 **Gibbs traces of identical transcripts**. Gibbs traces for two transcripts that have identical sequences, ENST00000436491 and ENST00000415119, and their sums. The individual transcript estimates exhibit high variability and anti-correlation, but the total expression level of the two transcripts can be well estimated. Click here for file

Additional file 2 **Poisson regression coefficients for three lanes in the HapMap dataset**. Plots of the Poisson regression coefficients obtained using the method described in [8] from three lanes in the HapMap dataset. The first two plots are for two lanes of the same Illumina GAII run (3125_2 and 3125_7), while the last plot is for a lane in a separate run (3122_7). The coefficients are highly stable across both lanes and runs. Click here for file

Additional file 3 **Plots of adjusted transcript lengths**. Scatterplot of log10 true vs. adjusted transcript lengths (top) and histogram of the log10 fold change in transcript length after adjustment (bottom). The adjustments are in general very slight. Click here for file

Additional file 4 **Transcript connectivity bar plot**. Bar plot of the number of transcripts that each transcript is connected to via shared reads for human and mouse. Click here for file

Additional file 5 **MMSEQ vs. RSEM scatterplots**. Normalized simulated expression vs. log ratio between simulated and estimated normalized expression for RSEM (left) and MMSEQ GS (right) (note the difference in the scales of the y-axes). The RSEM estimates tend to underestimate some low-to-medium expression values and set them very close to zero, which translates to large negative log ratios. This also applies to MMSEQ EM estimates. The posterior means estimated using MMSEQ Gibbs sampling are less biased except for a slight upwards bias for very lowly expressed transcripts. Click here for file

Additional file 6 **Quantile-quantile plots between pairs of lanes of the same individual and between pairs of lanes of different individuals**. Quantile-quantile plots of transcript expression estimates between pairs of lanes in the HapMap dataset. The lane IDs are shown along the diagonal. The bottom-left triangle shows pair-wise comparisons for a single individual sequenced in seven lanes of the same run. The upper-right triangle shows pair-wise comparisons between different individuals all sequenced in different lanes. There is a striking contrast in the consistency of the distribution of high values between pairs in the two triangles. Click here for file

Additional file 7 **Log-base mean-variance correlation between technical and biological replicates**. Scatterplots of log mean expression values against the log of the variance across technical and biological replicates at the transcript and gene levels. Each scatterplot has a line with a gradient of one if it shows technical replicates and two if it shows biological replicates. The variance is approximately proportional to the mean for technical replicates and the square of the mean for biological replicates. Click here for file

Additional file 8 **Scatterplots of log expression estimates from individual and pooled data**. Left: scatterplot of log expression estimates of male NA12045 vs. NA12872 obtained from individual datasets. Center: scatterplot of log expression estimates of male NA12045 obtained from the individual vs. pooled data. Right: scatterplot of log expression estimates of male NA12872 obtained from the individual vs. pooled data. Click here for file

Additional file 9 **Reciprocal vs. initial cross, omitting transcripts on the X chromosome**. Scatterplot of log fold changes between haplo-isoforms in the reciprocal (F_1_r) and the initial (F_1_i) cross, omitting transcripts on the X chromosome. Click here for file

Additional file 10 **Reciprocal vs. initial cross, highlighting isoforms containing at least one significant SNP**. Scatterplot of log fold changes between haplo-isoforms in the reciprocal (F_1_r) and the initial (F_1_i) cross, highlighting in green circles and red crosses isoforms containing at least one significant SNP imbalanced towards the paternal and maternal strain respectively. SNPs were called significant using a *χ*^2 ^goodness-of-fit test with a *P*-value threshold of 0.05 and are listed in [2]. Some transcripts contain significant SNPs with opposing imbalances, one example of which is clearly visible in the bottom-right quadrant. Click here for file

## References

[B1] McManus CJ Coolon JD Duff MO Eipper-Mains J Graveley BR Wittkopp PJ Regulatory divergence in Drosophila revealed by mRNA-seq. Genome Res 2010 20 816 825 10.1101/gr.102491.109 20354124 PMC2877578

[B2] Gregg C Zhang J Weissbourd B Luo S Schroth GP Haig D Dulac C High-resolution analysis of parent-of-origin allelic expression in the mouse brain. Science 2010 329 643 648 10.1126/science.1190830 20616232 PMC3005244

[B3] Mortazavi A Williams BA McCue K Schaeffer L Wold B Mapping and quantifying mammalian transcriptomes by RNA-Seq. Nat Methods 2008 5 621 628 10.1038/nmeth.1226 18516045 PMC13303166

[B4] Richard H Schulz MH Sultan M Nürnberger A Schrinner S Balzereit D Dagand E Rasche A Lehrach H Vingron M Haas SA Yaspo ML Prediction of alternative isoforms from exon expression levels in RNA-Seq experiments. Nucleic Acids Res 2010 20150413 10.1093/nar/gkq041 PMC2879520

[B5] Jiang H Wong WH Statistical inferences for isoform expression in RNA-Seq. Bioinformatics 2009 25 1026 1032 10.1093/bioinformatics/btp113 19244387 PMC2666817

[B6] Li B Ruotti V Stewart RM Thomson JA Dewey CN RNA-Seq gene expression estimation with read mapping uncertainty. Bioinformatics 2010 26 493 500 10.1093/bioinformatics/btp692 20022975 PMC2820677

[B7] Hansen KD Brenner SE Dudoit S Biases in Illumina transcriptome sequencing caused by random hexamer priming. Nucleic Acids Res 2010 38 e131 10.1093/nar/gkq224 20395217 PMC2896536

[B8] Li J Jiang H Wong WH Modeling non-uniformity in short-read rates in RNA-Seq data. Genome Biol 2010 11 R50 10.1186/gb-2010-11-5-r50 20459815 PMC2898062

[B9] Trapnell C Williams BA Pertea G Mortazavi A Kwan G van Baren MJ Salzberg SL Wold BJ Pachter L Transcript assembly and quantification by RNA-Seq reveals unannotated transcripts and isoform switching during cell differentiation. Nat Biotechnol 2010 28 511 515 10.1038/nbt.1621 20436464 PMC3146043

[B10] Degner JF Marioni JC Pai AA Pickrell JK Nkadori E Gilad Y Pritchard JK Effect of read-mapping biases on detecting allele-specific expression from RNA-sequencing data. Bioinformatics 2009 25 3207 3212 10.1093/bioinformatics/btp579 19808877 PMC2788925

[B11] Heap GA Yang JHM Downes K Healy BC Hunt KA Bockett N Franke L Dubois PC Mein CA Dobson RJ Albert TJ Rodesch MJ Clayton DG Todd JA van Heel DA Plagnol V Genome-wide analysis of allelic expression imbalance in human primary cells by high-throughput transcriptome resequencing. Hum Mol Genet 2010 19 122 134 10.1093/hmg/ddp473 19825846 PMC2792152

[B12] Fontanillas P Landry CR Wittkopp PJ Russ C Gruber JD Nusbaum C Hartl DL Key considerations for measuring allelic expression on a genomic scale using high-throughput sequencing. Mol Ecol 2010 19 Suppl 1 212 227 10.1111/j.1365-294X.2010.04472.x 20331781 PMC3217793

[B13] Novocraft. http://novocraft.com

[B14] Bayesian Gene eXpression. http://bgx.org.uk

[B15] Goncalves A Tikhonov A Brazma A Kapushesky M A pipeline for RNA-seq data processing and quality assessment. Bioinformatics 2011 21233166 10.1093/bioinformatics/btr012 PMC3051320

[B16] Montgomery SB Sammeth M Gutierrez-Arcelus M Lach RP Ingle C Nisbett J Guigo R Dermitzakis ET Transcriptome genetics using second generation sequencing in a Caucasian population. Nature 2010 464 773 777 10.1038/nature08903 20220756 PMC3836232

[B17] Trapnell C Pachter L Salzberg SL TopHat: discovering splice junctions with RNA-Seq. Bioinformatics 2009 25 1105 1111 10.1093/bioinformatics/btp120 19289445 PMC2672628

[B18] Li H Handsaker B Wysoker A Fennell T Ruan J Homer N Marth G Abecasis G Durbin R 1000 Genome Project Data Processing Subgroup The sequence alignment/map format and SAMtools. Bioinformatics 2009 25 2078 2079 10.1093/bioinformatics/btp352 19505943 PMC2723002

[B19] Su SY Asher JE Jarvelin MR Froguel P Blakemore AIF Balding DJ Coin LJM Inferring combined CNV/SNP haplotypes from genotype data. Bioinformatics 2010 26 1437 1445 10.1093/bioinformatics/btq157 20406911 PMC2913665

[B20] Langmead B Trapnell C Pop M Salzberg SL Ultrafast and memory-efficient alignment of short DNA sequences to the human genome. Genome Biol 2009 10 R25 10.1186/gb-2009-10-3-r25 19261174 PMC2690996

[B21] Anders S Huber W Differential expression analysis for sequence count data. Genome Biol 2010 11 R106 10.1186/gb-2010-11-10-r106 20979621 20979621 PMC3218662

[B22] Law AM Confidence intervals in discrete event simulation: a comparison of replication and batch means. Naval Res Logist Q 1977 23 667 678 10.1002/nav.3800240414

[B23] UCSC Genome Browser. http://genome.ucsc.edu

[B24] Gregg C Zhang J Butler JE Haig D Dulac C Sex-specific parent-of-origin allelic expression in the mouse brain. Science 2010 329 682 685 10.1126/science.1190831 20616234 PMC2997643

[B25] Puck JM Willard HF X inactivation in females with X-linked disease. N Engl J Med 1998 338 325 328 9445416 10.1056/NEJM199801293380611

