# Supplementary figures and images for: Haplotype and isoform specific expression estimation using multi-mapping RNA-seq reads
Source: Genome Biol. 2011 Feb 10;12(2):R13. doi: 10.1186/gb-2011-12-2-r13 (PMC3188795; doi:10.1186/gb-2011-12-2-r13)

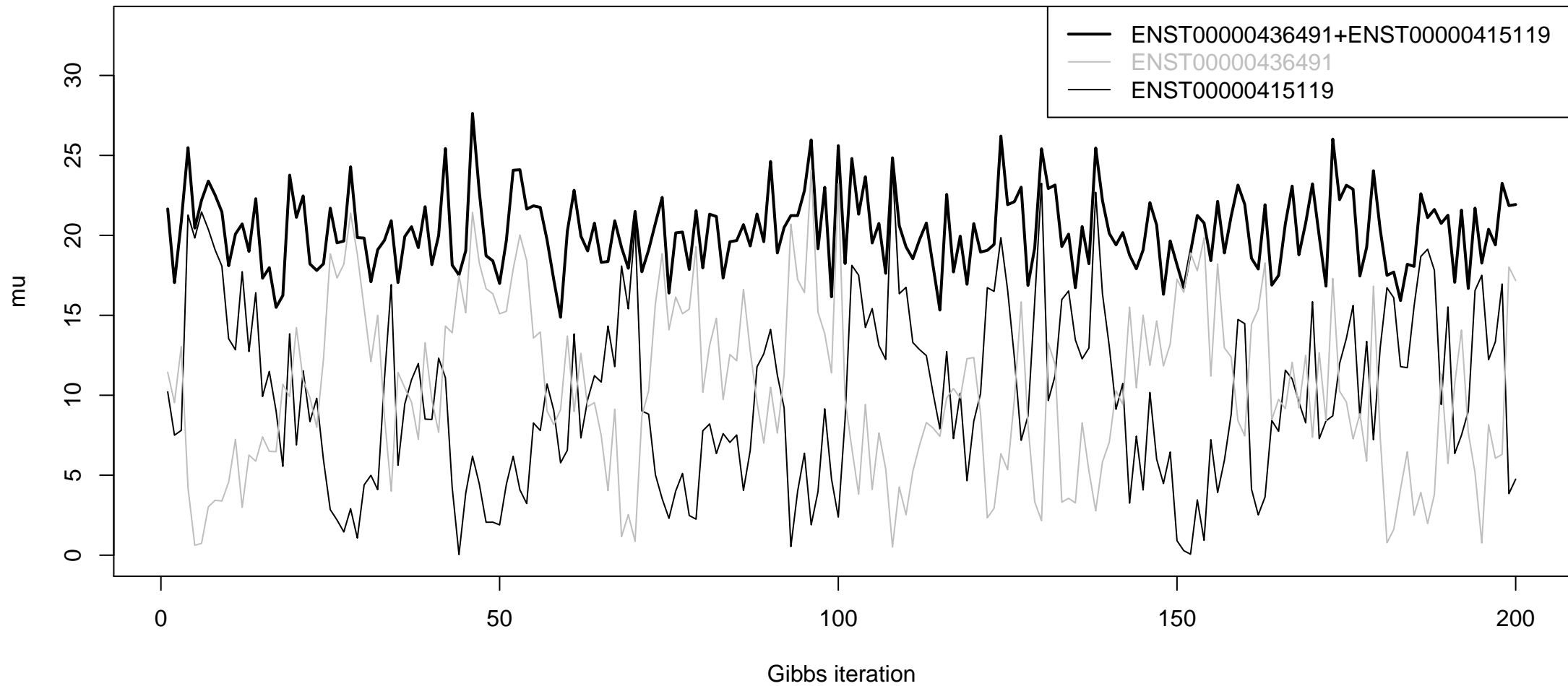

Supplement: Additional file 1 — Gibbs traces of identical transcripts. Gibbs traces for two transcripts that have identical sequences, ENST00000436491 and ENST00000415119, and their sums. The individual transcript estimates exhibit high variability and anti-correlation, but the total expression level of the two transcripts can be well estimated. [file gb-2011-12-2-r13-S1.PDF]

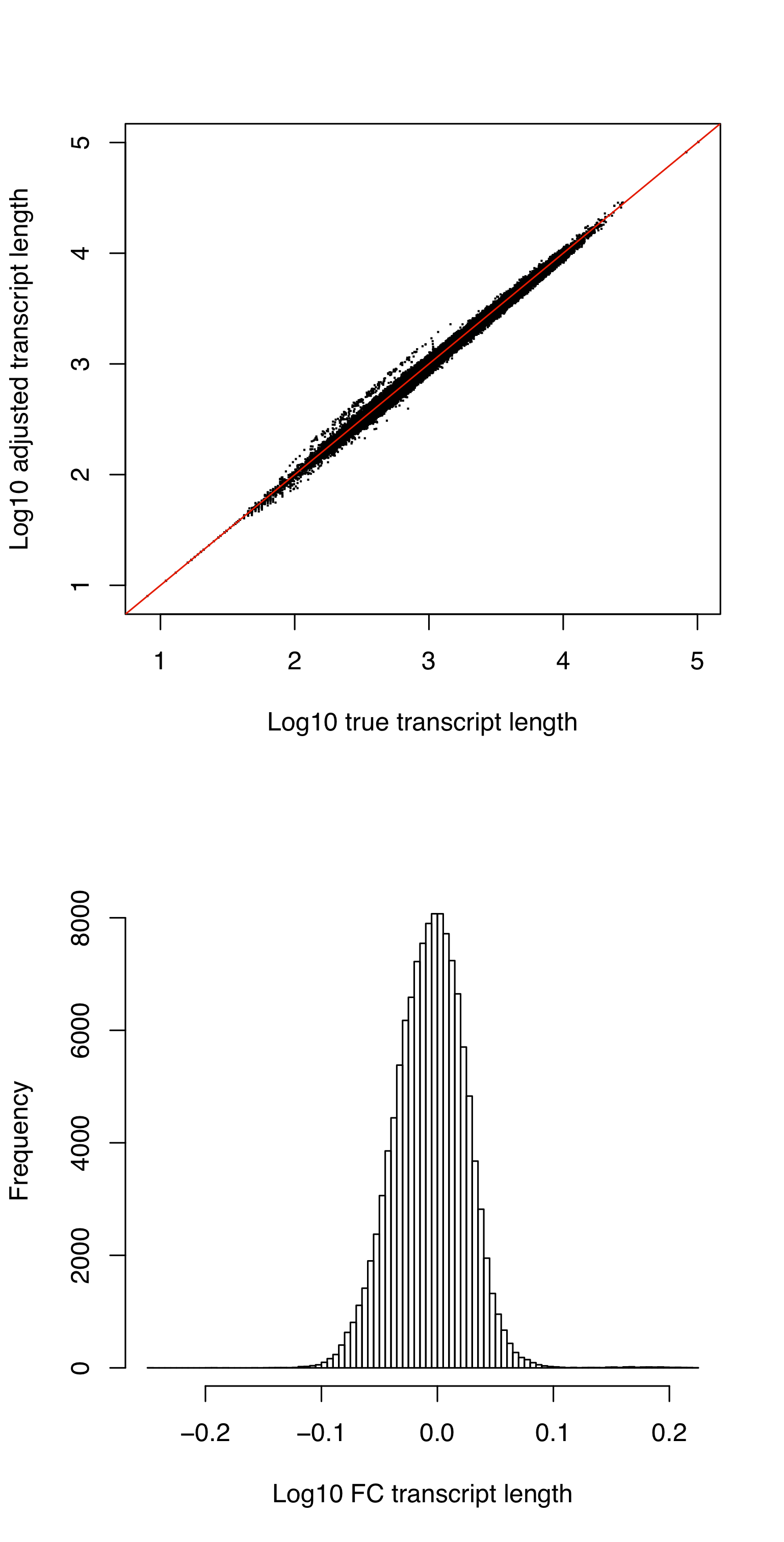

Supplement: Additional file 3 — Plots of adjusted transcript lengths. Scatterplot of log10 true vs. adjusted transcript lengths (top) and histogram of the log10 fold change in transcript length after adjustment (bottom). The adjustments are in general very slight. [file gb-2011-12-2-r13-S3.PNG]

## Human

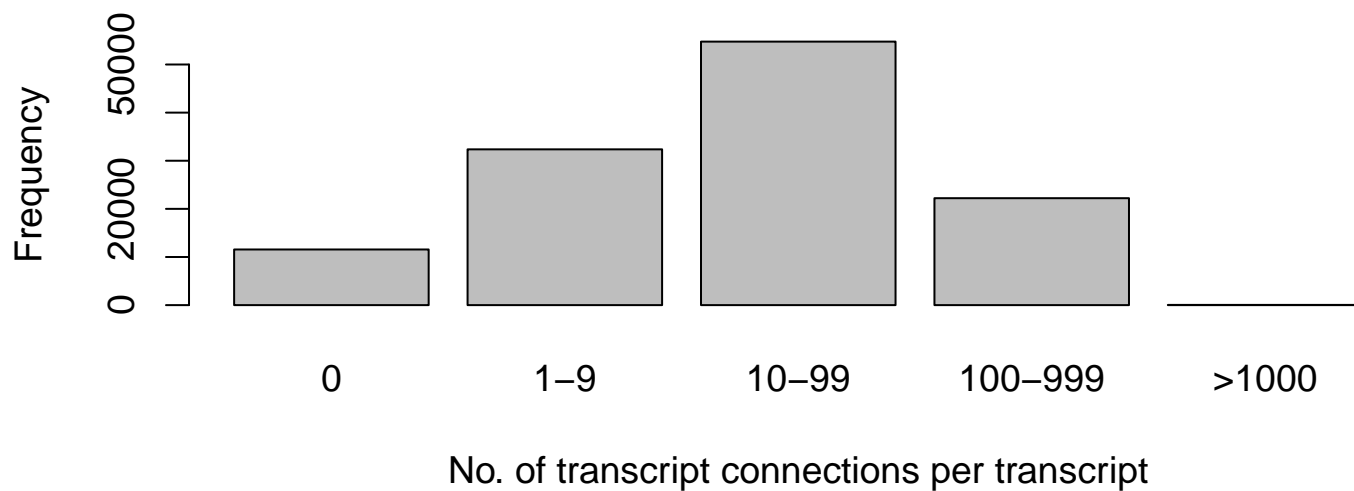

## Mouse

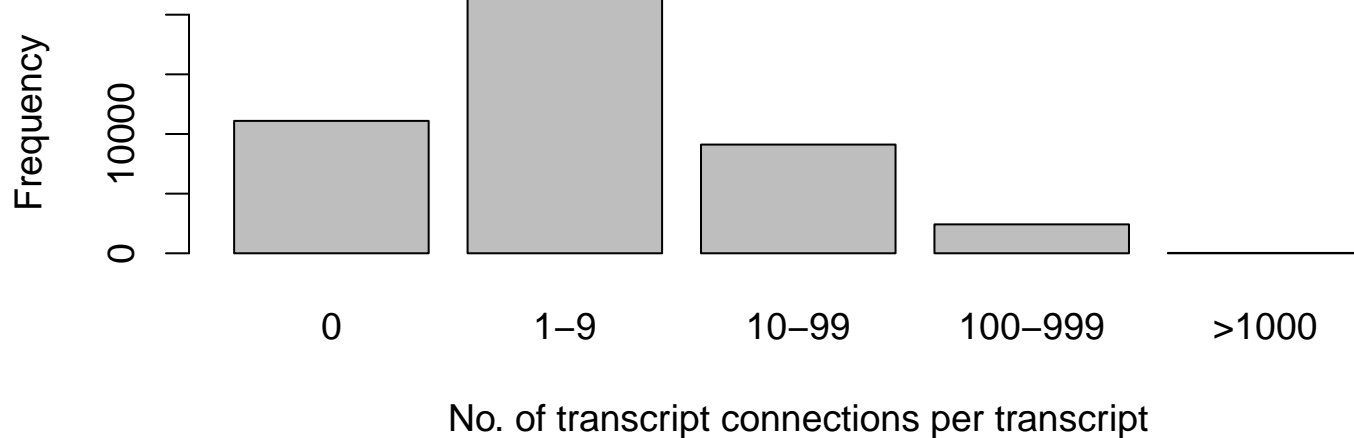

Supplement: Additional file 4 — Transcript connectivity bar plot. Bar plot of the number of transcripts that each transcript is connected to via shared reads for human and mouse. [file gb-2011-12-2-r13-S4.PDF]

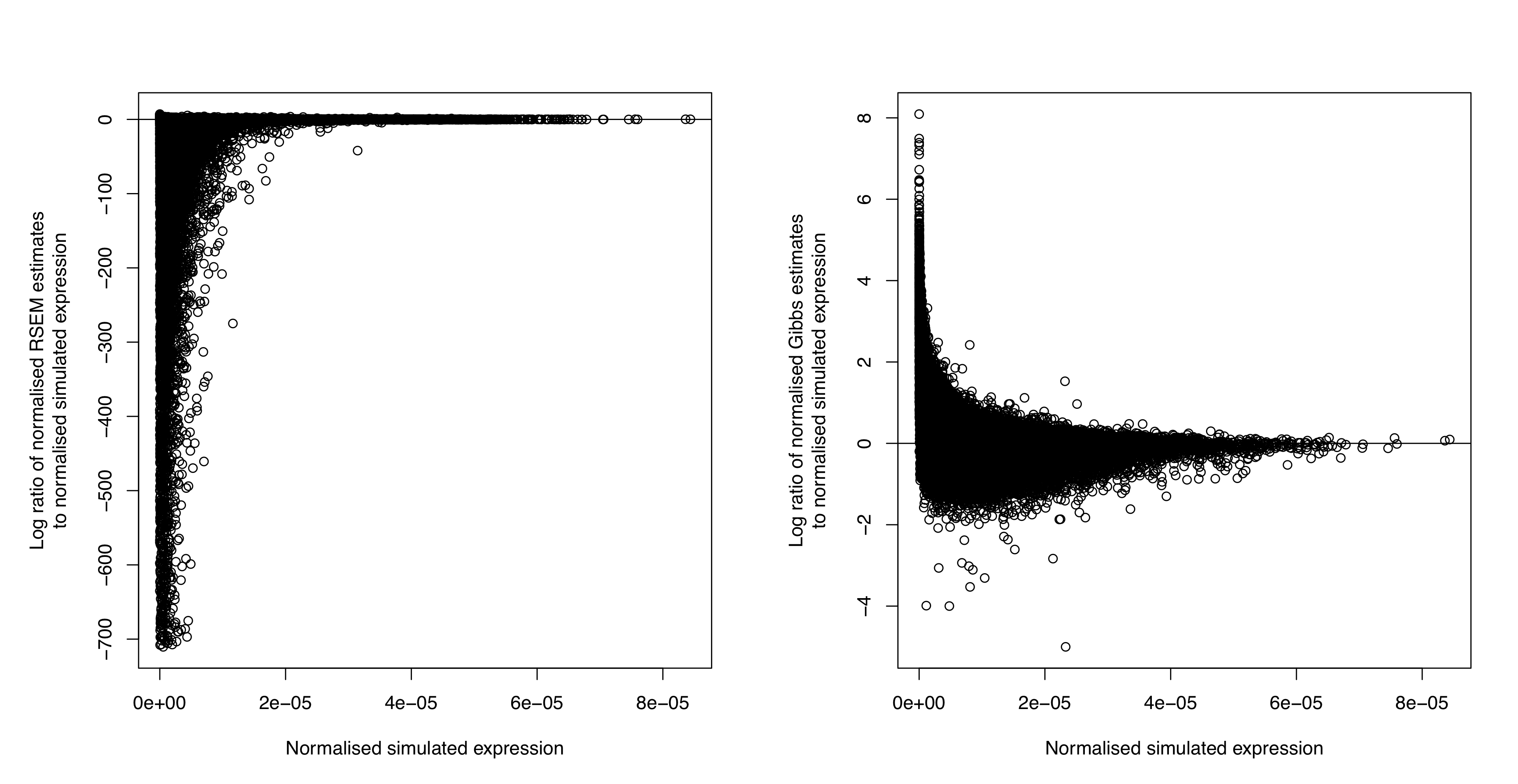

Supplement: Additional file 5 — MMSEQ vs. RSEM scatterplots. Normalized simulated expression vs. log ratio between simulated and estimated normalized expression for RSEM (left) and MMSEQ GS (right) (note the difference in the scales of the y-axes). The RSEM estimates tend to underestimate some low-to-medium expression values and set them very close to zero, which translates to large negative log ratios. This also applies to MMSEQ EM estimates. The posterior means estimated using MMSEQ Gibbs sampling are less biased except for a slight upwards bias for very lowly expressed transcripts. [file gb-2011-12-2-r13-S5.PNG]

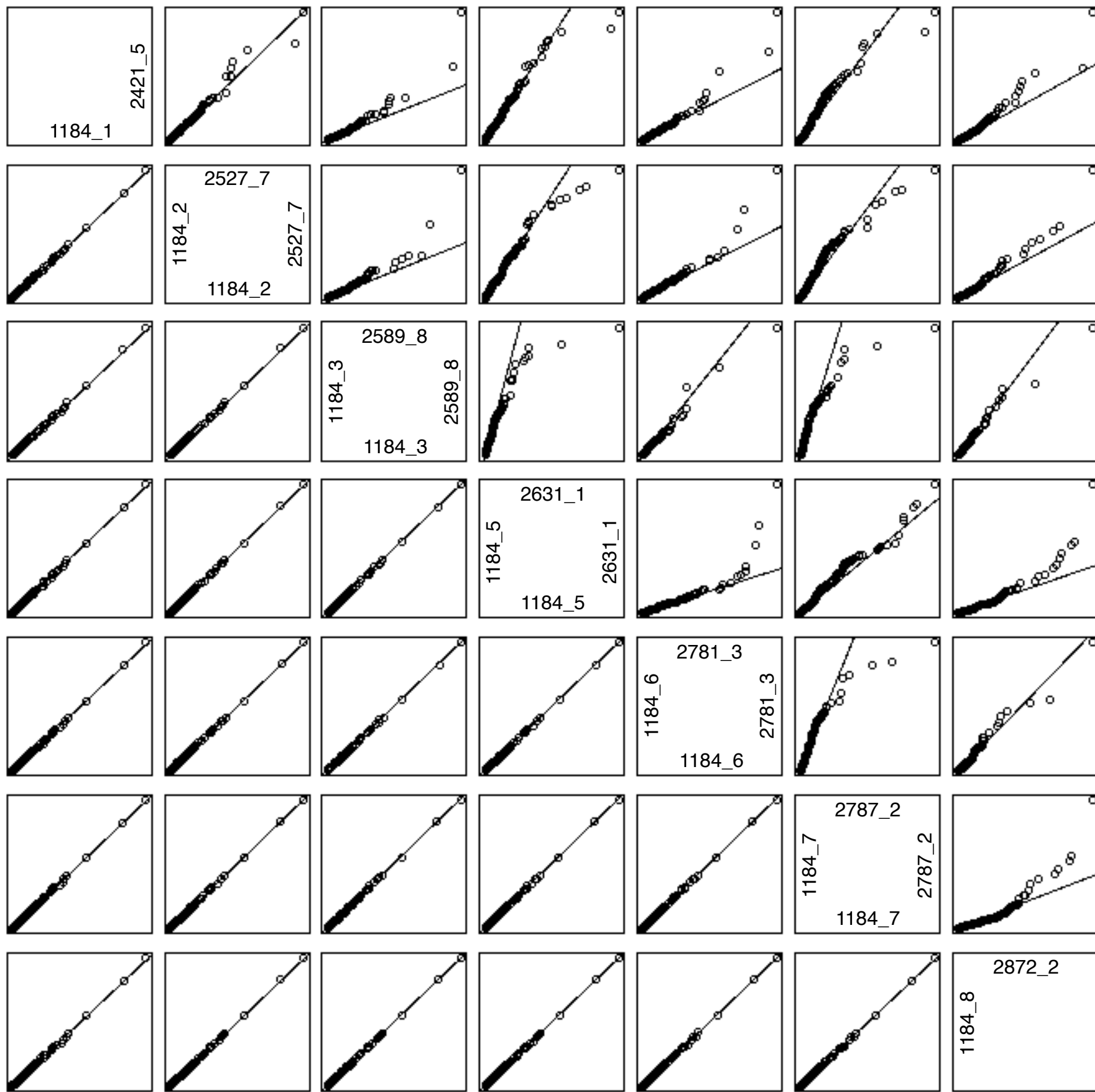

Supplement: Additional file 6 — Quantile-quantile plots between pairs of lanes of the same individual and between pairs of lanes of different individuals. Quantile-quantile plots of transcript expression estimates between pairs of lanes in the HapMap dataset. The lane IDs are shown along the diagonal. The bottom-left triangle shows pair-wise comparisons for a single individual sequenced in seven lanes of the same run. The upper-right triangle shows pair-wise comparisons between different individuals all sequenced in different lanes. There is a striking contrast in the consistency of the distribution of high values between pairs in the two triangles. [file gb-2011-12-2-r13-S6.PDF]

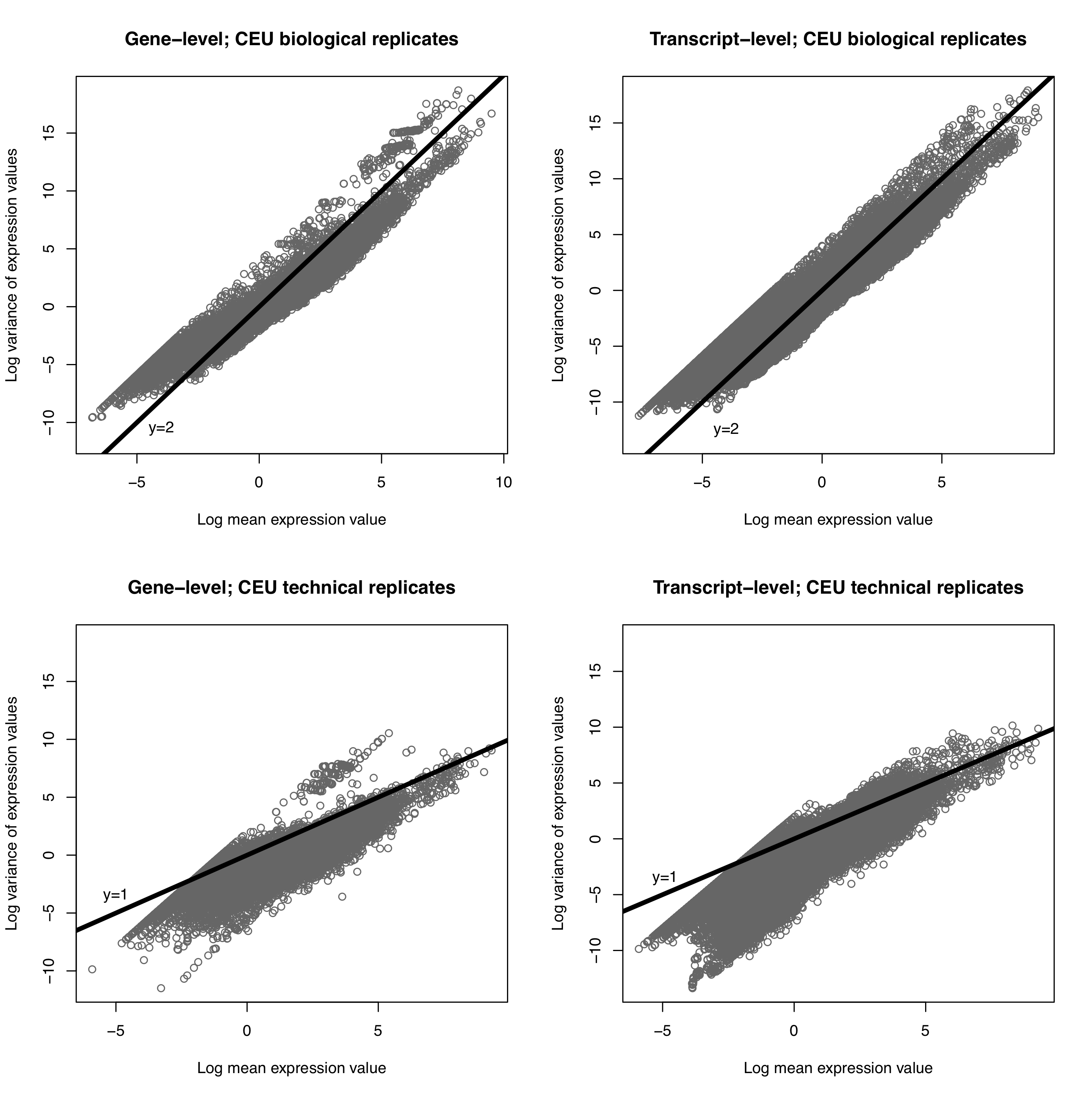

Supplement: Additional file 7 — Log-base mean-variance correlation between technical and biological replicates. Scatterplots of log mean expression values against the log of the variance across technical and biological replicates at the transcript and gene levels. Each scatterplot has a line with a gradient of one if it shows technical replicates and two if it shows biological replicates. The variance is approximately proportional to the mean for technical replicates and the square of the mean for biological replicates. [file gb-2011-12-2-r13-S7.PNG]

NA12872 estimates (individual data)

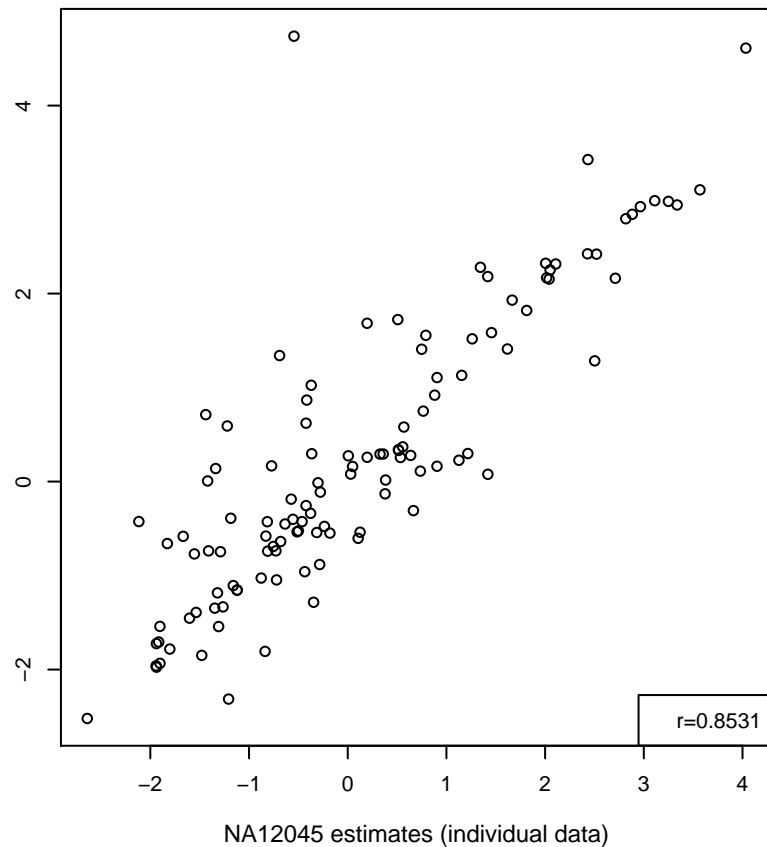

NA12045 estimates (pooled data)

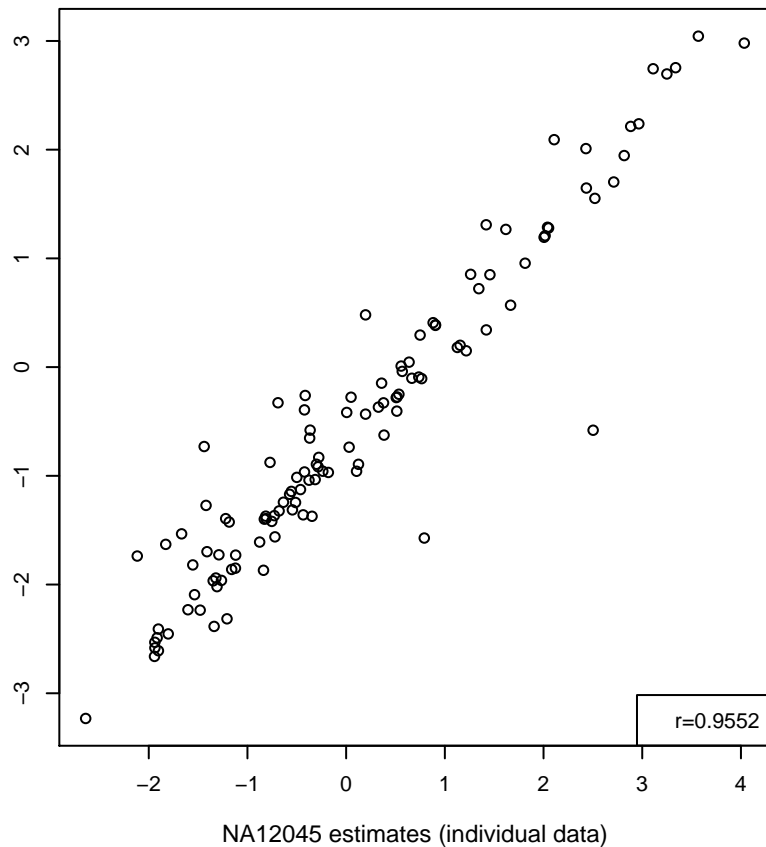

NA12872 estimates (pooled data)

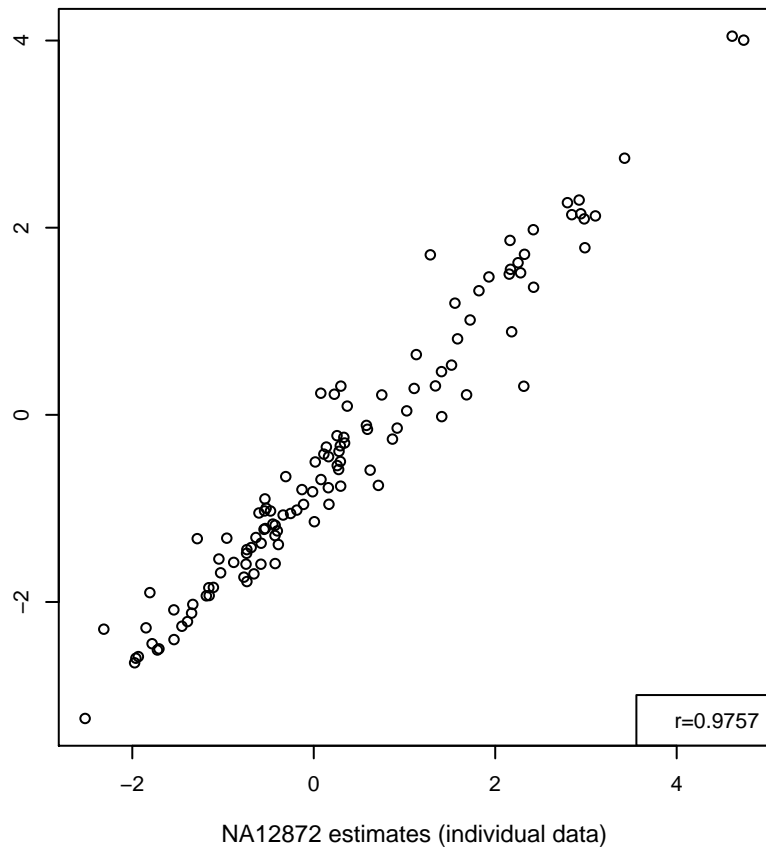

Supplement: Additional file 8 — Scatterplots of log expression estimates from individual and pooled data. Left: scatterplot of log expression estimates of male NA12045 vs. NA12872 obtained from individual datasets. Center: scatterplot of log expression estimates of male NA12045 obtained from the individual vs. pooled data. Right: scatterplot of log expression estimates of male NA12872 obtained from the individual vs. pooled data. [file gb-2011-12-2-r13-S8.PDF]

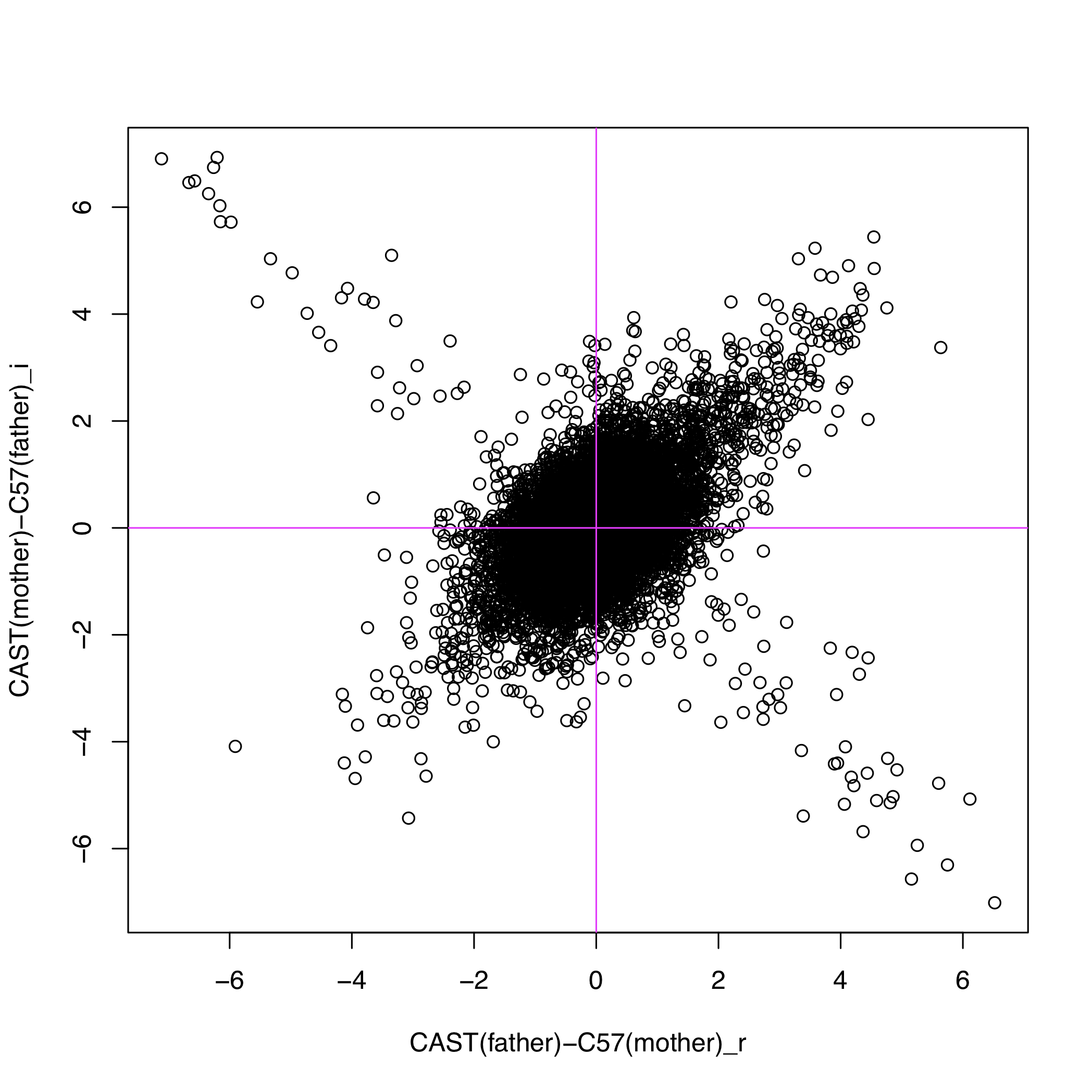

Supplement: Additional file 9 — Reciprocal vs. initial cross, omitting transcripts on the X chromosome. Scatterplot of log fold changes between haplo-isoforms in the reciprocal (F1r) and the initial (F1i) cross, omitting transcripts on the X chromosome. [file gb-2011-12-2-r13-S9.PNG]

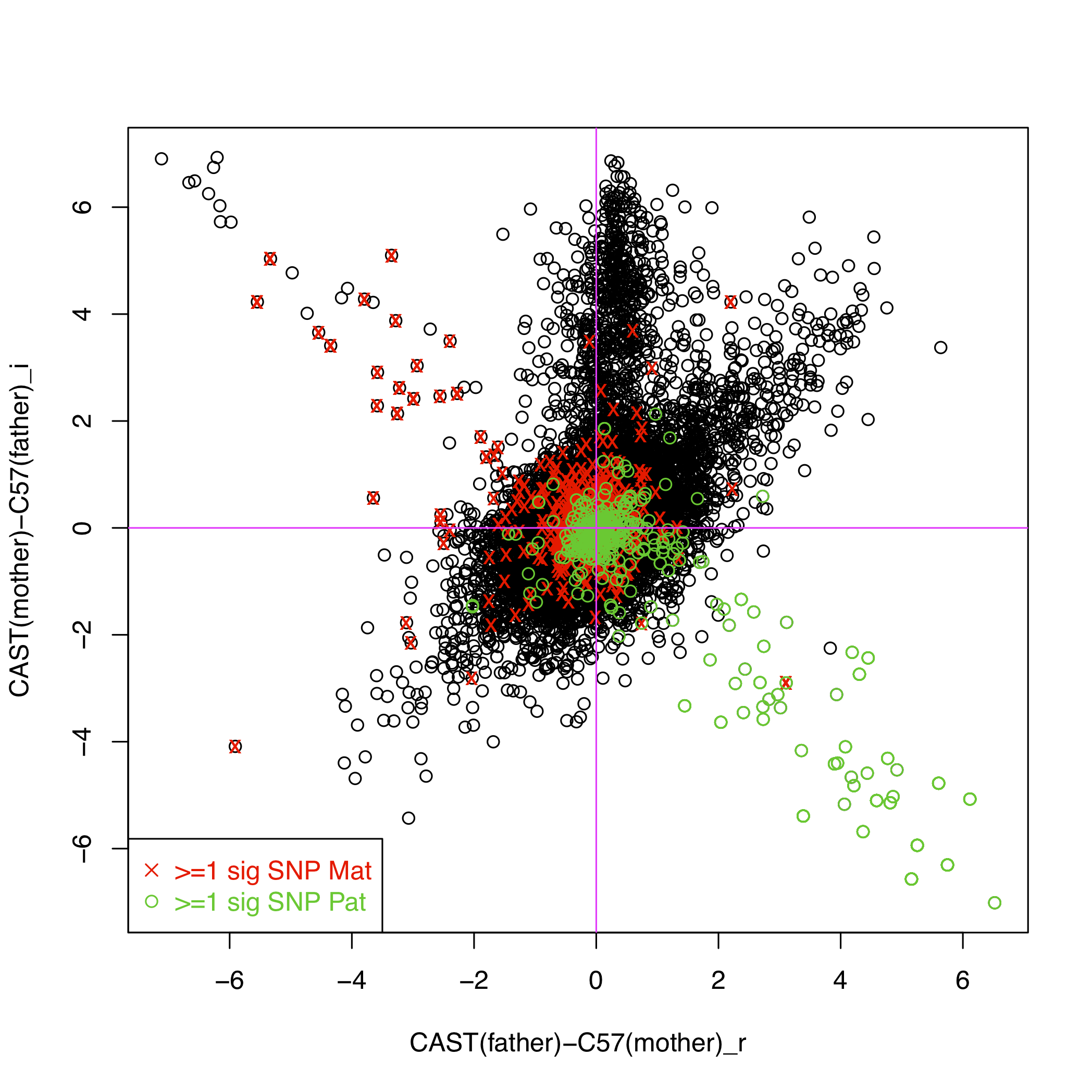

Supplement: Additional file 10 — Reciprocal vs. initial cross, highlighting isoforms containing at least one significant SNP. Scatterplot of log fold changes between haplo-isoforms in the reciprocal (F1r) and the initial (F1i) cross, highlighting in green circles and red crosses isoforms containing at least one significant SNP imbalanced towards the paternal and maternal strain respectively. SNPs were called significant using a χ2 goodness-of-fit test with a P-value threshold of 0.05 and are listed in [2]. Some transcripts contain significant SNPs with opposing imbalances, one example of which is clearly visible in the bottom-right quadrant. [file gb-2011-12-2-r13-S10.PNG]
