# Supplementary material for: Haplotype and isoform specific expression estimation using multi-mapping RNA-seq reads
Source: Genome Biol. 2011 Feb 10;12(2):R13. doi: 10.1186/gb-2011-12-2-r13 (PMC3188795; doi:10.1186/gb-2011-12-2-r13)

3125_2 coefficients, red−T, green−A, blue−C, black−G

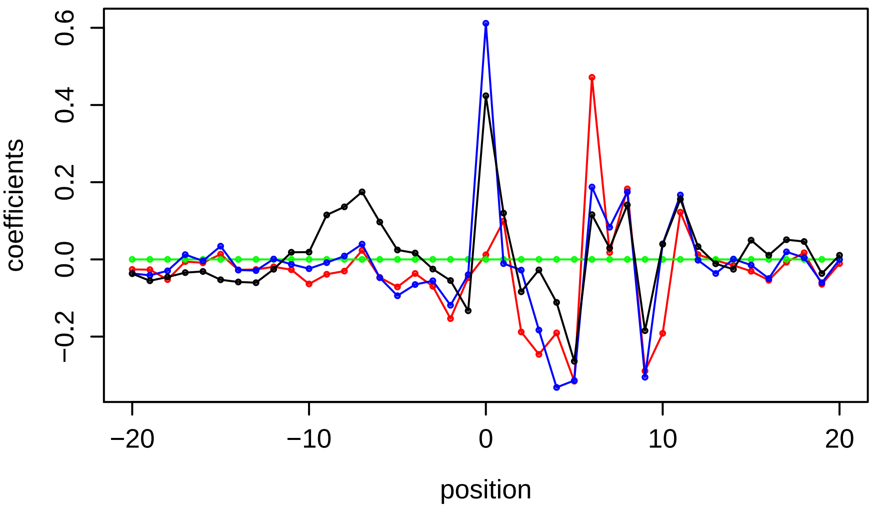

3125_7 coefficients, red−T, green−A, blue−C, black−G

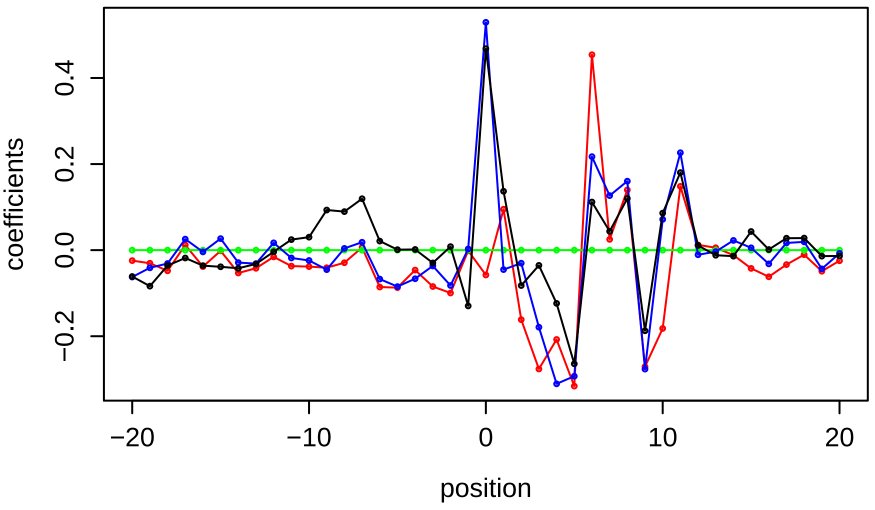

3122_7 coefficients, red−T, green−A, blue−C, black−G

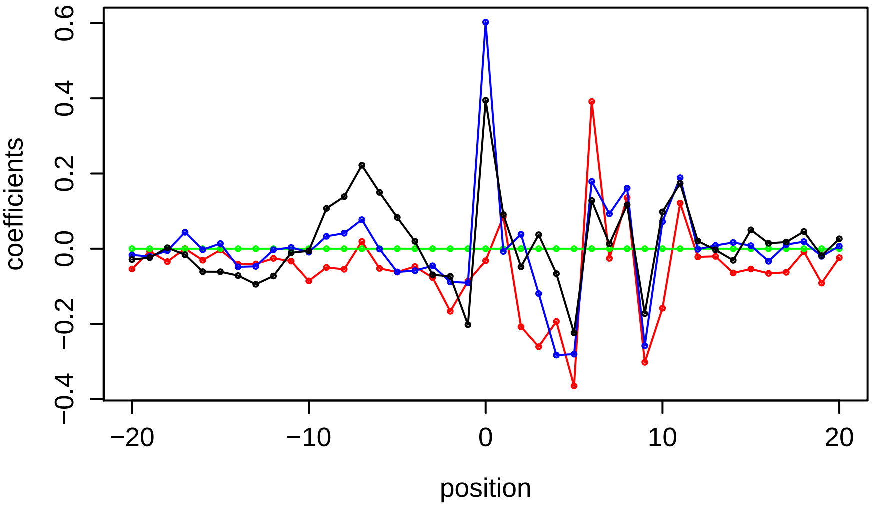

Supplement: Additional file 2 — Poisson regression coefficients for three lanes in the HapMap dataset. Plots of the Poisson regression coefficients obtained using the method described in [8] from three lanes in the HapMap dataset. The first two plots are for two lanes of the same Illumina GAII run (3125_2 and 3125_7), while the last plot is for a lane in a separate run (3122_7). The coefficients are highly stable across both lanes and runs. [file gb-2011-12-2-r13-S2.PDF]
